# Correlation of CRISPR/Cas and Antimicrobial Resistance in *Klebsiella pneumoniae* Clinical Isolates Recovered from Patients in Egypt Compared to Global Strains

**DOI:** 10.3390/microorganisms11081948

**Published:** 2023-07-30

**Authors:** Amany K. Alkompoz, Samira M. Hamed, Ahmed S. Abu Zaid, Thamer A. Almangour, Mohamed H. Al-Agamy, Khaled M. Aboshanab

**Affiliations:** 1 National Food Safety Authority (NFSA), Giza 11361, Egypt; amany.k7aled@gmail.com; 2 Department of Microbiology and Immunology, Faculty of Pharmacy, October University for Modern Sciences and Arts (MSA), 6th of October, Giza 12451, Egypt; satwa@msa.edu.eg; 3 Department of Microbiology and Immunology, Faculty of Pharmacy, Ain Shams University, Cairo 11566, Egypt; ahmed.abouzid@pharma.asu.edu.eg; 4 Department of Clinical Pharmacy, College of Pharmacy, King Saud University, Riyadh 11451, Saudi Arabia; talmangour@ksu.edu.sa; 5 Department of Pharmaceutics, College of Pharmacy, King Saud University, Riyadh 11451, Saudi Arabia; malagamy@ksu.edu.sa; 6 Department of Microbiology and Immunology, Faculty of Pharmacy, Al-Azhar University, Cairo 11651, Egypt

**Keywords:** antimicrobial resistance, CRISPR/Cas, *Klebsiella pneumoniae*, I-E, I-E*, plasmids, spacers, WGS

## Abstract

The CRISPR/Cas system has been long known to interfere with the acquisition of foreign genetic elements and was recommended as a tool for fighting antimicrobial resistance. The current study aimed to explore the prevalence of the CRISPR/Cas system in *Klebsiella pneumoniae* isolates recovered from patients in Egypt in comparison to global strains and correlate the CRISPR/Cas to susceptibility to antimicrobial agents. A total of 181 clinical isolates were PCR-screened for *cas* and selected antimicrobial resistance genes (ARGs). In parallel, 888 complete genome sequences were retrieved from the NCBI database for in silico analysis. CRISPR/Cas was found in 46 (25.4%) isolates, comprising 18.8% type I-E and 6.6% type I-E*. Multidrug resistance (MDR) and extensive drug resistance (XDR) were found in 73.5% and 25.4% of the isolates, respectively. More than 95% of the CRISPR/Cas-bearing isolates were MDR (65.2%) or XDR (32.6%). No significant difference was found in the susceptibility to the tested antimicrobial agents among the CRISPR/Cas-positive and -negative isolates. The same finding was obtained for the majority of the screened ARGs. Among the published genomes, 23.2% carried CRISPR/Cas, with a higher share of I-E* (12.8%). They were confined to specific sequence types (STs), most commonly ST147, ST23, ST15, and ST14. More plasmids and ARGs were carried by the CRISPR/Cas-negative group than others, but their distribution in the two groups was not significantly different. The prevalence of some ARGs, such as *bla*_KPC_, *bla*_TEM_, and *rmtB*, was significantly higher among the genomes of the CRISPR/Cas-negative strains. A weak, nonsignificant positive correlation was found between the number of spacers and the number of resistance plasmids and ARGs. In conclusion, the correlation between CRISPR/Cas and susceptibility to antimicrobial agents or bearing resistance plasmids and ARGs was found to be nonsignificant. Plasmid-targeting spacers might not be naturally captured by CRISPR/Cas. Spacer match analysis is recommended to provide a clearer image of the exact behavior of CRISPR/Cas towards resistance plasmids.

## 1. Introduction

The CRISPR/Cas system (Clustered Regularly Interspersed Short Palindromic Repeats (CRISPR)/CRISPR-associated genes (Cas)) is a genome component of prokaryotes that provides acquired immunity against foreign invaders [1]. It was unintentionally discovered in 1987 in the genome of *E. coli* [2] and was later found in approximately all archaeal genomes and in about half of the bacterial genomes, but it has not been found in any eukaryote so far [3].

A single or multiple CRISPR loci may exist in bacterial genomes [4]. Each locus of a fully functional CRISPR/Cas system consists of a CRISPR array, an upstream leader sequence, and a cluster of *cas* genes. The CRISPR array is made up of short direct repeats (22–56 nucleotides) interspaced by unique DNA fragments called “spacers”, whose origin is mobile genetic elements (MGEs), such as bacteriophages, plasmids, or transposons. Such spacers serve as sequence-specific memory of preceding infections and mediate the visibility of the invaders during subsequent infections. CRISPR arrays are transcribed into CRISPR RNA (crRNA) that guides Cas proteins, encoded by *cas* genes, to identify and destroy the invader’s foreign genetic elements. Finally, the leader sequence provides a promoter for the transcription of crRNA [5].

The CRISPR/Cas immune response works in three stages: adaptation, expression, and interference. During adaptation, a fragment of the intruder genome known as a protospacer is captured by the acquisition machinery (Cas1 and Cas2) and is integrated within the CRISPR array in the host chromosome to form a new spacer. A protospacer adjacent motif (PAM), which serves as a binding signal for Cas proteins, guides the selection of the protospacer. Using the RNA polymerase, the CRISPR array is transcribed into a pre-crRNA during the expression stage. This is further processed into a mature crRNA and cleaved to form individual small crRNAs, each representing a single spacer sequence. During the interference stage, crRNAs associate with an effector protein or combination of proteins to survey the cell for spacer sequence-complementary nucleic acid. If detected, the foreign DNA is cleaved or destroyed by the nuclease activity of the Cas protein [3,6]. Cas proteins are involved in all steps of CRISPR/Cas-mediated immunity, starting from new spacer integration and transcript processing to target nucleic acid cleavage [7].

The CRISPR/Cas system is grouped into two classes that were further categorized into six types and over forty subtypes. A multi-Cas effector protein complex is used by Class 1 members for interference, while Class 2 members use a single effector protein. Based on their signature Cas protein that cleaves foreign nucleic acids, these are divided into types. Types I, III, and IV belong to Class 1 CRISPR/Cas, while Class 2 comprises types II, V, and VI. The signature genes for different CRISPR/Cas types include *cas3* (type I), *cas9* (type II), *cas10* (type III), *csf1* (type IV), *cas12* (type V), and *cas13* (type VI). Based on the architecture of the CRISPR/Cas locus, CRISPR/Cas types are further divided into several subtypes [8].

CRISPR/Cas-based systems show promise as a cutting-edge strategy for developing novel antimicrobial agents that might help in the battle against multidrug resistant (MDR) pathogens [9]. They can be selectively utilized for inactivating genes involved in bacterial viability, pathogenicity, or virulence. They also provide an opportunity to combat antibiotic resistance in pathogens by either inactivating chromosomal genes or curing plasmids that encode antibiotic resistance [10]. In this context, many studies have been conducted with the aim of exploring the potential contribution of the native CRISPR/Cas system in the natural resistance of different bacterial species against the acquisition of resistance plasmids [11,12,13,14].

*Klebsiella pneumoniae* is an important Gram-negative opportunistic pathogen. It is a major contributor to many nosocomial infections, including urinary tract infections, pneumonia, neonatal sepsis, cystitis, endocarditis, and pyogenic liver abscesses [15]. The widespread dissemination of MDR, extensively drug-resistant (XDR), and hypervirulent (*hv*) strains of *K. pneumoniae* is associated with the acquisition of plasmids loaded with antibiotic resistance or hypervirulence genes [16]. Type I-E CRISPR/Cas was previously identified in the chromosome of *K. pneumoniae* [17,18,19], while type IV CRISPR was found on its plasmids [20]. On the basis of the allelic sequences of *cas1* and *cas3* genes, a subset of the type I-E CRISPR/Cas carried by *K. pneumoniae*, designated I-E*, was identified by [18]. Based on their location in the chromosome, CRISPR loci were also classified by [18] into CRISPR1 to 5. As CRISPR/Cas provides immunity against foreign DNA [5], its role in the dissemination of antimicrobial resistance plasmids and the evolution of MDR organisms has been long questioned in several bacterial species [13,21,22,23,24,25,26] and in *K. pneumoniae*, as well [19,27,28,29,30]. Nevertheless, a great discrepancy was noted in the conclusions drawn from previous research about the role of the CRISPR/Cas system (types I-E and I-E*) in the susceptibility of *K. pneumoniae* to antimicrobial agents. Moreover, reports about CRISPR/Cas in *K. pneumoniae* from the Middle East region are scarce, and the complete genomes from the region constitute only a minority of those deposited in the public databases.

The current study aimed to investigate the prevalence of CRISPR/Cas in *K. pneumoniae* clinical isolates obtained from patients admitted to Nasser Institute Hospital in Cairo, Egypt, and to study its association with antimicrobial resistance. In parallel, 888 *K. pneumoniae* published genomes were retrieved for comparison and further analysis. Additionally, the correlation between CRISPR/Cas and the number of resistance plasmids and ARGs was also assessed in the published genomes.

## 2. Materials and Methods

### 2.1. Collection of Clinical Isolates of Klebsiella pneumoniae

A total of 181 clinical *Klebsiella pneumoniae* isolates were obtained from the Microbiology Lab Nasser Institute Hospital, Cairo, Egypt. The obtained isolates were recovered from unidentified clinical specimens that were collected from patients for routine checkups in the period from April to September 2021. These were isolated from blood (n = 70, 39.0%), urine (n = 49, 27.0%), wound secreta (n = 33, 18.0%), CSF (n = 12, 6.6%), sputum (n = 11, 6.0%), and pus (n = 6, 3.3%). All isolates were identified by conventional biochemical tests and stored at −20 °C in Lauria Bertani (LB) broth containing 25% glycerol (*v*/*v*) until use. Isolate 117, selected for whole genome sequencing (WGS), was further identified using the matrix-assisted laser desorption/ionization–time of flight mass spectrometry (MALDI-TOF MS) technique and the WGS-based identification tool Speciesfinder (http://www.genomicepidemiology.org/) (accessed on 15 July 2023). The study was conducted in accordance with the ethical principles stated in the Declaration of Helsinki and was approved by the institutional ethics committee, Faculty of Pharmacy, Ain Shams University (ENREC-ASU-RHDIRB2020110301-63).

### 2.2. Molecular Screening of cas Genes and Selected ARGs

Extraction of the DNA of all bacterial isolates was conducted with the boiling method, as described before [31,32,33]. After suspending a few colonies of each isolate in sterile distilled water, the bacterial suspensions were boiled at 100 °C for 10 min, then centrifuged at a speed of 13,000 rpm for 5 min. The supernatant was then collected and stored at −20 °C until used. The signature genes of CRISPR/Cas, *cas1* and *cas3*, were screened in all isolates. As proposed by previous studies [18,19], two types of primers were used for screening *cas1* and *cas3* genes, each of which corresponds to a certain CRISPR/Cas type. Amplification of *cas1*/*cas3* using form A primers denotes the presence of CRISPR/Cas type I-E, while form B primers correspond to type I-E*. Multiplex and uniplex polymerase chain reactions (PCRs) were used to screen selected genes conferring resistance to carbapenems (*bla*_NDM_)**_,_** cephalosporins (*bla*_CTX-M_ and *bla*_SHV_), aminoglycosides (*aac(6’)-Ib* and *armA*), and fluoroquinolones (*qnrS*, *qnrB*, and *aac(6’)-Ib*). All amplification reactions were carried out according to the protocol described before [33], as follows: 95 °C for 5 min, then 30 cycles of 95 °C for 30 s, followed by 30 s at the annealing temperature (Ta) for each primer, then 1 min of extension at 72 °C, and finally, 10 min of final extension at 72 °C. PCR products were separated and detected by agarose gel electrophoresis using 1.5% agarose. The PCR primers used in the current study are listed in Table S1 (Supplementary File).

### 2.3. Antimicrobial Susceptibility Profiling

The antimicrobial susceptibility of the isolates to 17 antimicrobial agents was determined on Mueller–Hinton agar (Oxoid, UK) using the Kirby Bauer disc diffusion method, according to the guidelines of the Clinical and Laboratory Standards Institute (CLSI, 2020). The tested antimicrobial agents (Bioanalyse, Turkey) included Amoxicillin/clavulanate (30 µg), cefepime (30 µg), cefotaxime (30 µg), cefoxitin (30 µg), ceftriaxone (30 µg), ceftazidime (30 µg), aztreonam (10 µg), imipenem (10µg), amikacin (30 µg), gentamicin (10 µg), ciprofloxacin (5 µg), tetracycline (30 µg), trimethoprim 1.25 µg/sulphamethoxazole 23.75 µg (SXT, 25 µg), chloramphenicol (30 µg), nitrofurantoin (300 µg), and tigecycline (15 µg). The broth microdilution assay was used to determine the minimum inhibitory concentrations (MICs) of colistin. The breakpoints proposed by the European Committee on Antimicrobial Susceptibility Testing [34] were used for interpreting the antimicrobial susceptibility tests of tigecycline and colistin. MDR and XDR phenotypes were inferred according to the definitions of Magiorakos et al. [35].

### 2.4. Whole Genome Sequencing (WGS) of Representative Isolates Carrying Type I-E and Type I-E* CRISPR/Cas

The draft genomes of two *K. pneumoniae* isolates were used as positive controls for the PCR-based CRISPR/Cas screening. The draft genome of isolate K57 that carried type I-E CRISPR/Cas was generated in a previous study using an Illumina MiSeq sequencer (Illumina Inc., San Diego, CA, USA). Isolate 117 was used as a positive control for type I-E* CRISPR/Cas. The draft genome of 117 was generated in the current study using DNBseq technology. DNA was extracted using the Quick-DNA Fungal/Bacterial Miniprep Kit (Zymo Research, Irvine, CA, USA). Library preparation and WGS were conducted with BGI Tech Solutions Hong Kong Co., Ltd. (Hong Kong, China). WGS was performed on a DNBseq™ sequencing platform with 150 bp paired-end reads. Raw reads with low-quality sequences or adapter sequences were filtered using SOAPnuke software generated by BGI [36]. For de novo assembly of the filtered reads, Unicycler v0.4.8 was used. Annotation was automatically generated by the Prokaryotic Genome Annotation Pipeline (PGAP) [37] during submission to the National Center for Biotechnology Information genome submission portal (NCBI, https://submit.ncbi.nlm.nih.gov/)(accessed on 15 July 2023). The multilocus sequence types (MLSTs) of all strains were determined using the Pubmlst database (https://bigsdb.pasteur.fr/cgi-bin/bigsdb/bigsdb.pl?db=pubmlst_klebsiella_seqdef&page=sequenceQuery) (accessed on 15 July 2023). Plasmid sequences and plasmid replicons were identified using PlasmidFinder 2.1 [38] (https://cge.food.dtu.dk/services/PlasmidFinder/)(accessed on 15 July 2023) and the machine-learning-based webtool mplasmids v2.1.0 [39] (https://sarredondo.shinyapps.io/mlplasmids/)(accessed on 15 July 2023). The resistance gene identifier tool RGI 5.2.1 (https://card.mcmaster.ca/analyze/rgi) (accessed on 15 July 2023) was used to identify the ARGs. Both chromosomal and plasmid sequences of each strain were screened for CRISPR/Cas loci using the open-access webtool CRISPRFinder (https://crisprcas.i2bc.paris-saclay.fr/CrisprCasFinder/Index) (accessed on 15 July 2023). The nucleotide sequences of the CRISPR arrays were downloaded for further nBLAST analysis against the nt/nr nucleotide database using the Basic Local Alignment Search Tool (https://blast.ncbi.nlm.nih.gov/Blast.cgi) (accessed on 15 July 2023). CRISPR/Cas loci were visualized using SnapGene Viewer version 6.2.2.

### 2.5. Retrieval, Characterization, and CRISPR/Cas Analysis of Published Genomic Sequences of Klebsiella pneumoniae

In order to compare the distribution of the CRISPR/Cas system in our collection with other isolates collected worldwide, the same analysis was applied to *K. pneumoniae* genomes deposited in the genomic database of the NCBI (https://www.ncbi.nlm.nih.gov/genome/), accessed in 23 July 2021. A total of 888 complete genomes of *K. pneumoniae* were retrieved for in silico analyses, as described in Section 2.4.

### 2.6. Statistical Analyses

The statistical analyses were carried out using IBM SPSS statistics software version 26.0 (IBM Corp., Armonk, NY, USA) and GraphPad Prism 8 software (GraphPad Software, San Diego, CA, USA), with *p*-values of less than 0.05 considered statistically significant. Categorical variables were compared using the chi-square and Fisher’s exact tests. Numerical data were tested for normality, and those not normally distributed were compared using the Mann–Whitney U test, and the medians were used for describing their central tendency. For correlation analyses, the Spearman Rho correlation coefficient was calculated.

### 2.7. Data Availability

The draft genome of the I-E-positive isolate K57 is available in the GenBank database under the BioProject number PRJNA844012. The draft genome of the I-E*-positive isolate 117 generated in the current study was submitted to the GenBank database under the BioProject number PRJNA992508.

## 3. Results

### 3.1. Prevalence of cas Genes in the Collected Isolates

**Figure 1:**
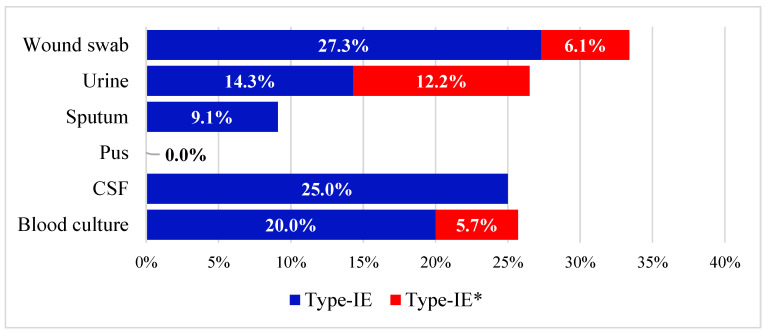
Stacked bar chart representing the distribution of type I-E and type I-E* CRISPR/Cas among different clinical specimen types.

The CRISPR/Cas signature genes, *cas* genes, were found in 25.4% of the tested isolates. According to the sequence of *cas1*/*cas3* genes, 34 (18.7%) isolates carried type I-E, while 12 (6.6%) were positive for type I-E*. All *cas1*-positive isolates were also *cas3*-positive. *K. pneumoniae* isolated here were recovered from patients with BSI (39%), UTI (27%), RTI (6%), CSF (7%), and wound infections (21%). While *cas* genes are more prevalent in blood and urine cultures compared to others, no significant difference was found in their distribution in the isolates that belong to different specimen types (Figure 1). Deeper analysis of the distribution of type I-E and type I-E* CRISPR/Cas in various specimen types revealed a higher prevalence for type I-E in all specimen types than type I-E*. Interestingly, the observed number of type I-E* urinary isolates (n = 6) was higher than the expected count (n = 3.2), but this was also statistically nonsignificant (Pearson’s chi-square *p*-value = 0.140).

### 3.2. Antibiotic Susceptibility Profiles and Correlation to cas Genes

**Figure 2:**
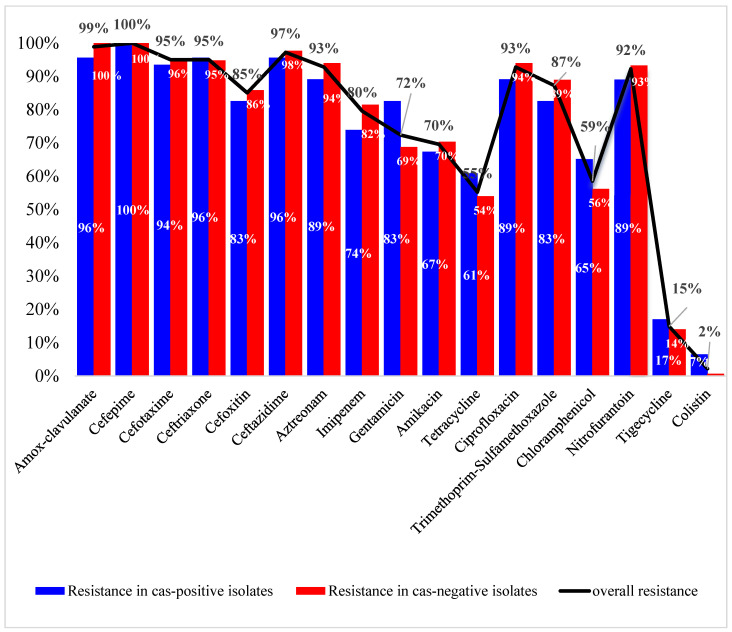
The prevalence of resistance to the tested antimicrobial agents in all isolates, *cas*-positive isolates, and *cas*-negative isolates. No significant association was found between non-susceptibility to any of the tested antimicrobials and carrying the *cas* genes.

Of all tested antimicrobial agents, the highest activity was shown by colistin (R = 2%), tigecycline (R = 15%), tetracycline (R = 55%), and chloramphenicol (R = 59%). Almost all isolates (99%) were non-susceptible to amoxicillin/clavulanate. The resistance to cephalosporins ranged from 85 to 100%, with cefepime and cefoxitin showing the lowest and highest activity, respectively. More than 90% of the isolates were resistant to aztreonam (93%), ciprofloxacin (93%), and nitrofurantoin (92%). High resistance levels were also found for trimethoprim-sulfamethoxazole (87%), the aminoglycoside gentamicin (72%), and amikacin (70%). Carbapenem resistance was phenotypically evident in 80% of the isolates. The observed resistance to most of the tested antimicrobial agents was less prevalent in the *cas*-positive isolates compared to others, as shown in Figure 2. Exceptions included gentamicin, tetracycline, chloramphenicol, tigecycline, and colistin. The correlation between antimicrobial resistance and CRISPR/Cas was nonsignificant (*p*-values are shown in Table S2).

**Figure 3:**
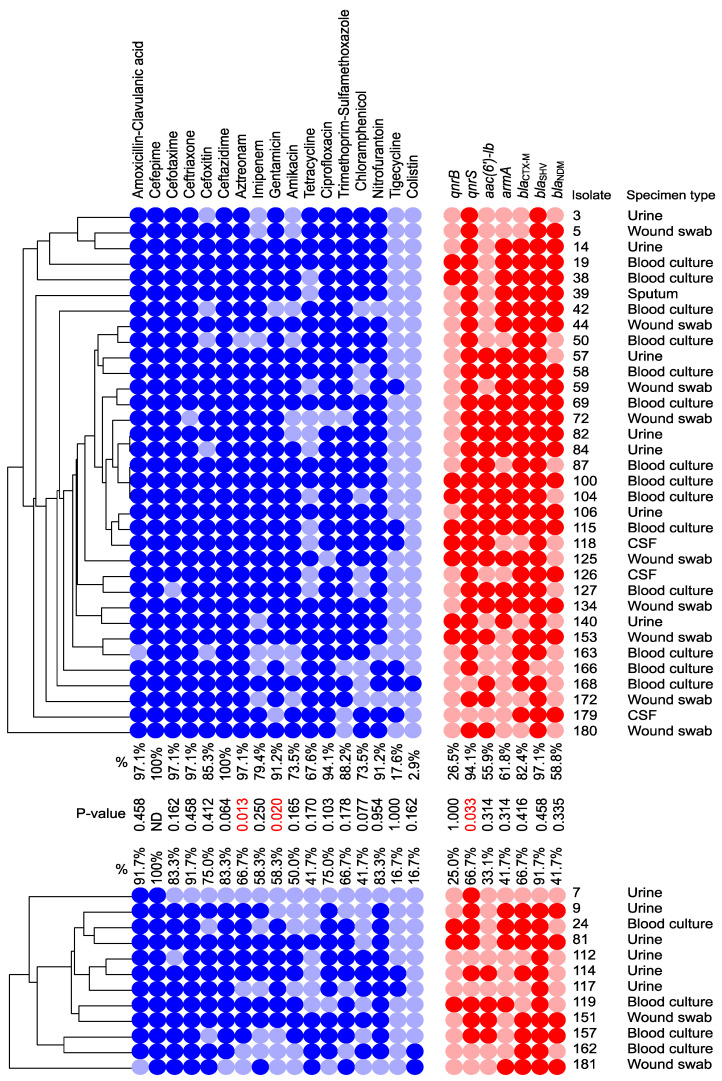
A heatmap displaying the antimicrobial resistance profiles and genetic determinants among the isolates carrying CRISPR/Cas type I-E (**Upper** panel) versus those carrying type I-E* (**Lower** panel); Red and pink colors indicate the presence and absence of the ARGs, respectively; Dark and light blue colors indicate phenotypic resistance and susceptibility to the tested antimicrobial agents, respectively. *p*-values are calculated using the chi-square or Fisher’s exact test, where appropriate. *p*-values in red color denote significant difference.

Higher resistance was found for all antimicrobial agents, except colistin, among the group of isolates carrying I-E compared to those that carried I-E* (Figure 3). This was significant only for aztreonam and gentamicin (Fisher’s exact test, *p*-values = 0.013 and 0.020, respectively). A total of 133/181 isolates (73.5%) were MDR, while 46/181 (25.4%) showed the XDR phenotype. Out of 133 MDR strains, 31 were CRISPR/Cas-positive. These comprised 21 I-E CRISPR/Cas and 10 I-E* CRISPR/Cas. Furthermore, among the XDR isolates, 13/46 carried I-E, and 2 carried I-E*.

### 3.3. Distribution of the ARGs and Correlation to cas Genes

**Figure 4:**
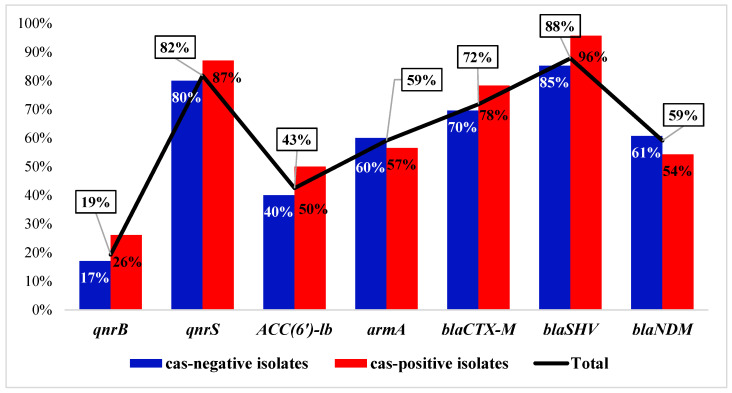
Prevalence of various ARGs and their distribution among the CRISPR/Cas-positive and CRISPR/Cas-negative groups. No significant association was found between any of the tested ARGs and CRISPR/Cas.

Of all the tested ARGs, *bla*_SHV_ and *qnrS* were found to have the highest prevalence. With respect to CRISPR/Cas, a higher prevalence was found for most of the ARGs in the *cas*-positive group compared to others (Figure 4). The observed prevalence was even higher than the values expected by the chi-square test. Exceptions included the *bla*_NDM_ and *armA* genes, which were more prevalent in the *cas*-negative isolates. No significant difference was found in the distribution of the ARGs across the two groups. Upon examination of the number of ARGs carried by the isolates in each group, we found that the *cas*-positive isolates carried more combinations of ARGs (Median = 5) compared to the *cas*-negative group (Median = 4), but this was not statistically significant, according to the Mann–Whitney U test (*p*-value = 0.070).

Upon analysis of the distribution of the ARGs in the groups of isolates carrying I-E versus those carrying I-E* CRISPR/Cas, we found a higher prevalence for all genes in the IE-positive isolates compared to the I-E*-positive ones (Figure 3). This was statistically significant only for *qnrS,* which was found in 94% of the IE-positive isolates versus 67% of the I-E*-positive isolates, based on Fisher’s exact test (*p*-value = 0.033).

### 3.4. Draft Genome Analysis of Representative Isolates Carrying I-E and I-E* CRISPR/Cas

**Figure 5:**
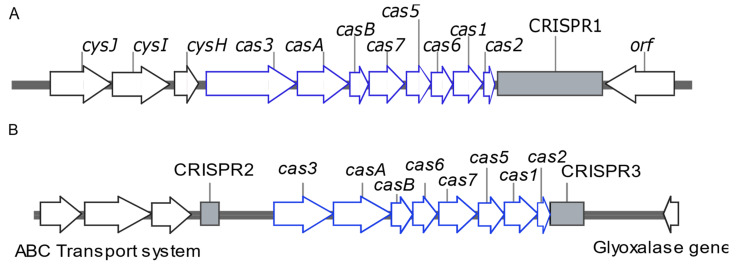
Architecture of CRISPR/Cas loci carried by the type I-E-positive isolate K57 (**A**) and the type I-E*-positive isolate 117 (**B**). Blue arrows represent cas loci, while other open reading frames are drawn in black. CRISPR arrays are represented by grey rectangles. The figure is not drawn to scale and was created by SnapGene Viewer version 6.2.2.

The genome sequence of *K. pneumoniae* isolate 117 was submitted to the NCBI GenBank database under the accession code SUB13646323. Isolate K57 carried one CRISPR array (CRISPR1), while Isolate 117 carried two CRISPR arrays, including CRISPR2 and CRISPR3. Only those identified with level 4 evidence were considered. The CRISPR array of K57 enclosed 50 spacers. Of them, only four (8.0%) spacers matched plasmid sequences, and seven (14.0%) spacers showed no similarity in the NCBI non-redundant nucleotide (nr/nt) database. Likewise, only one spacer harbored by one of the CRISPR arrays of isolate 117 matched plasmid sequences. The detailed features of the CRISPR/Cas system identified in K57 and 117 isolates are shown in Table S3, while the architecture of the CRISPR/Cas loci carried by the two strains is illustrated in Figure 5.

While only one plasmid replicon was identified in K57, six replicons were found in 117. Contigs identified as plasmids using both PlasmidFinder and mplasmids were used as input for RGI. None of the contigs identified by PlasmidFinder were found to carry ARGs, while those identified by the mplasmids tool carried the ARGs shown in Table 1. More ARGs were carried by the I-E*-positive isolate 117.

### 3.5. Features of the CRISPR/Cas Systems of the Published K. pneumoniae Genomes

In silico screening of 888 published *K. pneumoniae* genomes for the CRISPR/Cas system showed that only 206/888 (23.2%) harbored complete CRISPR/Cas systems. While at least one CRISPR array was found in 26.0% of the genomes, only those that also carried *cas* gene clusters were considered to have a functional CRISPR/Cas system and were selected for further analysis. In silico PCR analysis of the genomes using *cas*-specific primers showed that type I-E* (114/888, 12.8%) was more prevalent than type I-E (92/888, 10.4%). No matching was found for only three strains. The three CRISPR arrays co-existed in only 2/206 (1.0%) strains, while 55.3% and 43.7% carried two and three CRISPR arrays, respectively.

Four aspects of the CRISPR/Cas system were analyzed in the published genomes. These included the ST distribution, correlation with plasmids, correlation with ARGs, and spacer features.

#### 3.5.1. ST Distribution of the CRISPR/Cas-Positive Strains

**Figure 6:**
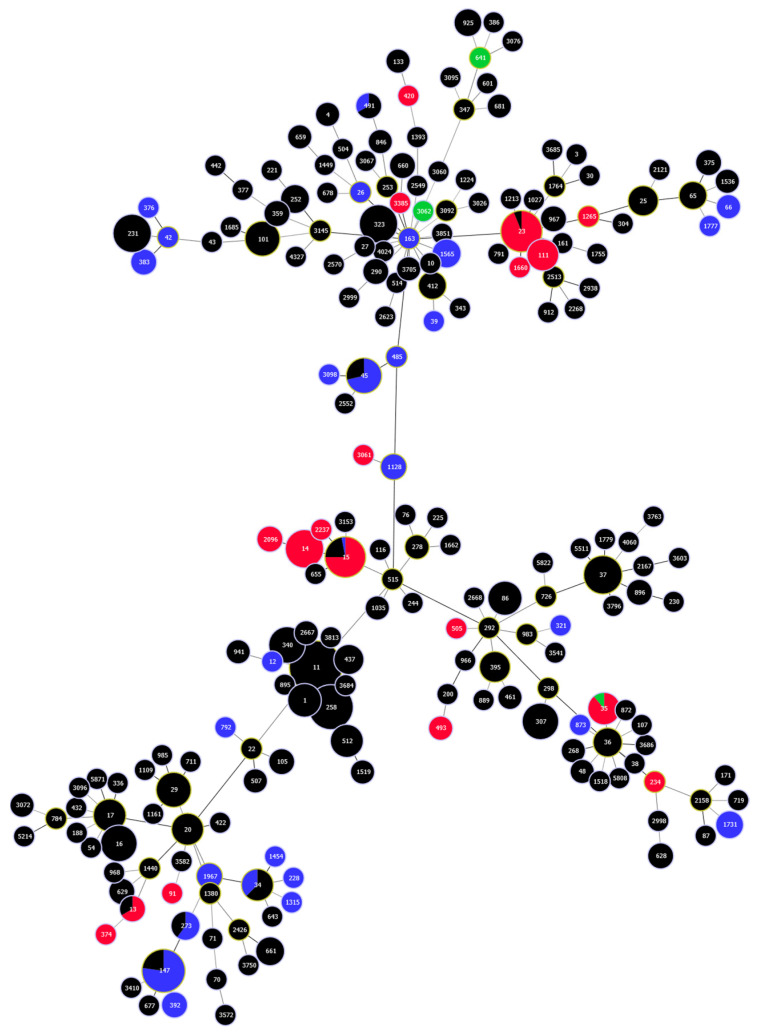
Minimum spanning tree generated using the STs assigned to the genomes investigated in the current study shows the distribution of CRISPR/Cas. The size of the circles corresponds to the number of isolates that belong to each ST; STs represented by black circles correspond to the CRISPR/Cas-free strains; CRISPR/Cas-positive strains carrying types I-E and I-E* are denoted by blue and red circles, respectively; strains carrying CRISPR/Cas systems not belonging to IE or I-E* are represented by green color. The figure was generated by Phyloviz software v2.0.

CRISPR/Cas systems were confined to 46 (24.0%) out of 191 STs assigned to the studied genomes. Most frequently, CRISPR/Cas was found in ST147 (16.5%), ST23 (15.0%), ST15 (12.1%), and ST14 (10.2%). Less frequently, it was found in other STs, as shown in Figure 6. I-E CRISPR/Cas was found in 28 STs, most commonly in ST147 (37.0%), ST45 (10.9%), and ST1565 (5.4%), while I-E* was distributed over 18 STs and prevailed in ST23 (27.9%), ST15 (21.6%), and ST14 (18.9%). Interestingly, I-E and I-E* CRISR/Cas systems were found in different STs, except for ST15, which harbored both IE-positive and I-E*-positive strains. A great diversity was also found in the number of spaces carried by the isolates that belonged to the same ST. While ST11 was the major ST, identified in 18.5% of the analyzed genomes, all ST11 strains were CRISPR/Cas-free. No CRISPR/Cas systems were found in any of the other STs that belong to the high-risk Clonal complex CC258 that includes ST258, ST340, and ST512. The STs of the 17 strains that carried full CRISPR/Cas with additional CRISPR arrays on their plasmids were also investigated. The STs of only 16 strains could be successfully determined. Of these, ten strains belonged to ST14; three strains belonged to ST147; and the remaining three strains belonged to ST15, ST209, and ST383.

#### 3.5.2. Correlation between CRISPR/Cas Systems and Plasmids Distribution

**Figure 7:**
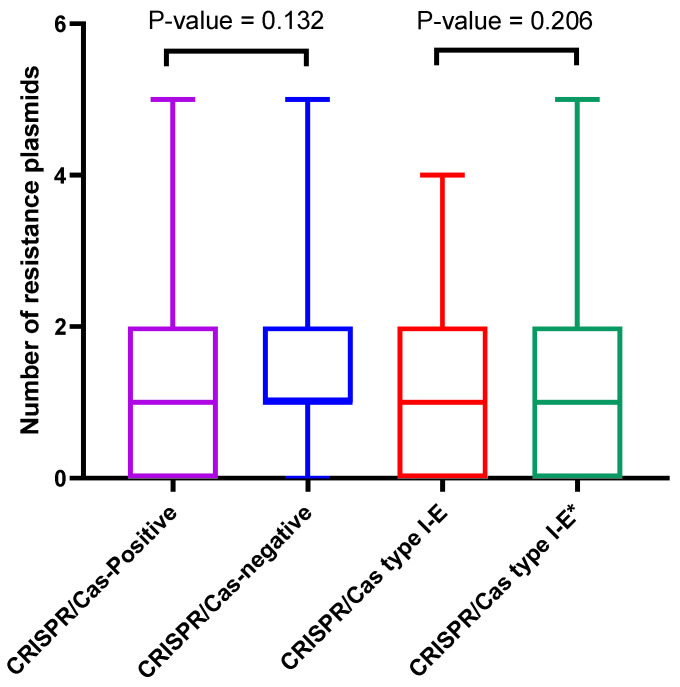
Box whisker plot illustrating the distribution of the resistance plasmids among CRISPR/Cas-positive and -negative strains and among the strains carrying type I-E versus type I-E* CRISPR/Cas.

Analysis of the distribution of the resistance plasmids with respect to the CRISPR/Cas system showed that at least one resistance plasmid was found in 75.8% of the CRISPR/Cas-negative strains versus 69.4% of those carrying CRISPR/Cas (chi-square, *p*-value = 0.066). The difference in the number of resistance plasmids across the two groups (medians = 1) was found to be nonsignificant, based on the Mann–Whitney U test (*p*-value = 0.132), as shown in Figure 7.

With respect to the CRISPR/Cas type, at least one resistance plasmid was carried by 73.9% of the I-E-positive strains versus 66.7% of those carrying I-E* (chi-square, *p*-value = 0.262). The difference in the number of resistance plasmids across the two groups was found to be nonsignificant (Mann–Whitney U test, *p*-value = 0.209). The value of the median was 1 for both groups.

Interestingly, CRISPR/Cas elements were also found on the plasmids of some strains. Plasmids of eight strains carried *cas3* genes in the absence of other *cas* genes or CRISPR arrays on their chromosomes. Furthermore, CRISPR arrays were also found on the plasmids of 33 strains. Of these, 17/33 (51.5%) also carried full CRISPR/Cas systems on their chromosomes. The majority (13/17; 76.5%) had the replicon type [IncHI1B, IncFIB], while other replicon types found included [IncFIA, IncR, IncFIB, IncHI1B], [IncHI1B, IncFIB, IncN], [IncFIB(K), IncFII(K)], and [IncHI1B], each of which was found in one plasmid. Fifteen plasmids (88.2%) also carried ARGs (Table S4).

Surprisingly, the number of ARGs and resistance plasmids carried by the strains that coharbored chromosomal and plasmid CRISPR arrays was significantly higher than that of those that carried chromosomal CRISPRs only. The median number of ARGs and plasmids was 12 and 2, respectively, in the former group versus 5 and 1, respectively, in the latter group. The *p*-values of the Mann–Whitney U test were <0.001 and 0.001 for ARGs and resistance plasmids, respectively.

#### 3.5.3. Correlation between CRISPR/Cas and ARGs

At least 57 ARGs conferring resistance to all classes of antimicrobials were identified by the RGI webtool, using plasmid sequences as input. The total number of plasmid-encoded resistance determinants was calculated and compared within the groups of strains that carried CRISPR/Cas versus others. At least one resistance gene was carried by 71.4% of the CRISPR/Cas-positive strains versus 76.0% of others, but the distribution of the ARGs in the two groups was not significantly different (Medians = 5 and 7, respectively; Mann–Whitney U test *p*-value = 0.358). Similarly, 72.8% of the I-E-positive strains carried at least one resistance gene compared to 70.3% of the I-E*-positive strains, but there was no significant difference in the distribution of the genes within the two groups (Medians = 6 and 4, respectively; Mann–Whitney U test *p*-value = 0.583).

Variable results were found for the distribution of individual genes within the groups of isolates carrying CRISPR/Cas systems compared to others (Table S5). Some genes were less prevalent in the presence of CRISPR/Cas systems, such as *bla*_TEM_, *bla*_KPC_, *bla*_LAP-2_, *rmtB*, *catA3*, and *fosA*, while others were not (e.g., *bla*_NDM_, *bla*_VIM_, *armA*, *mcr3*, *tet*(B), and *msrE*).

#### 3.5.4. CRISPR/Cas Spacers and Correlation to Resistance Plasmids

**Figure 8:**
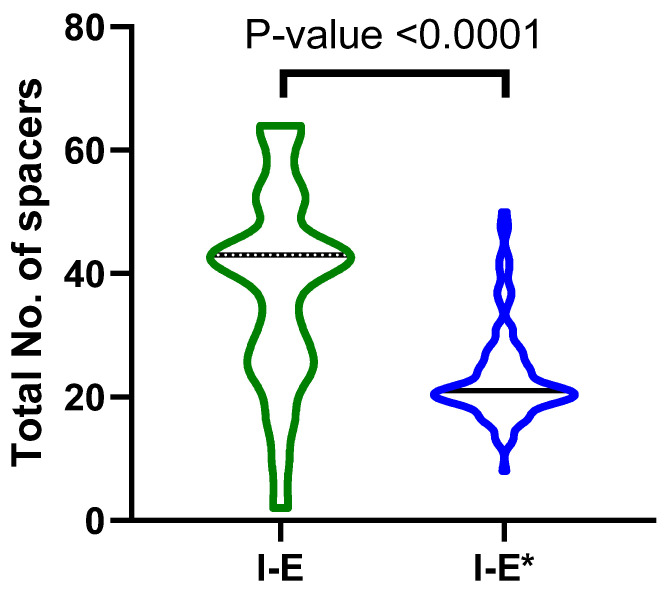
Violin plot showing the distribution of the spacers in the three types of CRISPR (A) and in the isolates carrying CRISPR/Cas IE compared to I-E*.

The total number of spacers carried by the isolates ranged from 2 to 64, with a median of 25 spacers. The median number of spacers carried by I-E was significantly higher (43) than that of I-E* (21). The distribution of spacers across the two groups is shown in Figure 8. Unexpectedly, a weak positive correlation was found between the total number of spacers and the total number of resistance plasmids carried by the CRISPR/Cas-positive isolates (Spearman’s Rho correlation coefficient = 0.033, *p*-value = 0.633). The same finding was obtained for the correlation between the number of spacers and the total number of ARGs (Spearman’s Rho correlation coefficient = 0.004, *p*-value = 0.949).

## 4. Discussion

Several publications have recently linked the presence of CRISPR/Cas systems to enhanced susceptibility to antibiotics and impeded plasmid acquisition in several bacterial species. Meanwhile, little research is being conducted in the Middle East to figure this out. In the current study, we aimed to investigate the prevalence and impact of the CRISPR/Cas system in one of the most challenging bacterial pathogens, *K. pneumoniae*. This was then compared to global strains, represented by their published genomes, in which other aspects of the CRISPR/Cas system were also explored.

Two *cas* genes were used for PCR screening of CRISPR/Cas systems. These included *cas1*, a universal *cas* gene that is found in all CRISPR/Cas types, and *cas3*, the signature gene of the type I CRISPR/Cas system [7]. PCR screening of the mentioned *cas* genes in a collection of 181 clinical isolates of *K. pneumoniae* revealed a CRISPR/Cas prevalence of 25.4%. This is consistent with previous reports from China that found CRISPR/Cas in 23.5% [40], 21.3% [29], and 14.9% [41] of their collections. CRISPR/Cas type I was previously classified into I-E and I-E* based on *cas1* and *cas3* alleles and their different locations in the chromosome. Type I-E is located in the *iap*-*cysH* region, and type I-E* is located in the ABC transport system–glyoxalase region [1,18,42]. Here, we found type I-E to have a higher prevalence (18.7%) than type I-E* (6.6%); similar findings were reported from Taiwan [19]. Contrarily, I-E* prevailed in the *K. pneumoniae* isolates studied by others [40,41]. Unlike the finding of Hu et al., IE and I-E* did not co-exist in any of the tested isolates or the analyzed published genomes [40]. While type I-E was found to have a higher prevalence than type I-E* in the isolates recovered from all specimen types, the observed number of urinary isolates that carried type I-E* was higher than the expected count calculated by the chi-square test. Conflicting findings were reported by Li et al., suggesting that there is no obvious relation between CRISPR/Cas and specific specimen types [19].

Most of our isolates were resistant to the majority of the tested antimicrobial agents, except colistin (2%) and tigecycline (15%). MDR and XDR phenotypes were found in 73.5% and 25.4% of the isolates, respectively. The high prevalence of MDR and XDR phenotypes was commonly reported in *K. pneumoniae* globally [42,43,44,45,46] and in Egypt [10,47,48]. Furthermore, a relatively low resistance was recorded against tetracycline (55%) and chloramphenicol (59%), considering these antibiotics can become promising alternatives for colistin and tigecycline against MDR and XDR *K. pneumoniae* strains, as recommended before [15,47]. As for the CRISPR/Cas-positive isolates, 45/46 (97.8%) were MDR (65.2%) or XDR (32.6%), and variable results were found for the susceptibility to different antimicrobial agents among the CRISPR/Cas-positive and CRISPR/Cas-negative strains. The same or higher resistance percentages were found for all β-lactams among the CRISPR/Cas-negative isolates compared to others. The same finding was evident for amikacin, ciprofloxacin, nitrofurantoin, and trimethoprim/sulfamethoxazole. Meanwhile, lower resistance was recorded for gentamicin, tetracycline, chloramphenicol, tigecycline, and colistin. This was not significant for any of the tested agents. Similar findings, but with statistical significance, were also reported by Wang et al., except for tetracycline [29]. Contrary to other results, the same study recorded complete susceptibility to carbapenems and ciprofloxacin among the CRISPR/Cas-positive isolates. Similarly, another study in the US reported that all *K. pneumoniae* strains with CRISPR/Cas were sensitive to carbapenems, as well as all other tested antibiotics [27], which contradicts the high carbapenem resistance revealed in the CRISPR/Cas-bearing isolates in the current study. Comparing the association of I-E and I-E* CRISPR/Cas with antimicrobial resistance, we found higher resistance to all tested antimicrobial agents among the isolates that carried I-E compared to those with I-E*; the only exception was colistin. In contrast, a previous study conducted on 176 clinical isolates from Taiwan showed that I-E was associated with lower resistance to gentamicin, trimethoprim/sulfamethoxazole, and cefotaxime, compared to I-E* [19]. Ref. [41] found no significant difference in the antimicrobial susceptibility among the CRISPR/Cas-negative and CRISPR/Cas-positive isolates carrying I-E and I-E*.

The prevalence of seven ARGs was PCR-tested in all our isolates. The extended-spectrum β-lactamase (ESBL)-coding genes *bla*_SHV_ and *bla*_CTX-M_, as well as the plasmid-encoded quinolone resistance gene *qnrS*, were found to have a high prevalence. A lower prevalence of *qnrS* was previously reported from Egypt [49,50]. Less frequently, the isolates carried the carbapenemase-coding gene *bla*_NDM_ and the aminoglycoside resistance genes *armA* and *aac(6’)-Ib*, while the gene *qnrB* was the least prevalent. In agreement with our findings, low prevalence was previously reported for *qnrB* in Egypt (17%) [50] and Tunisia (13%) [51]. Surprisingly, most of the tested ARGs were more frequently found in the CRISPR/Cas-positive isolates. An exception was found for *bla*_NDM_ and *armA*, which was also reflected in the higher resistance to imipenem and amikacin among the CRISPR/Cas-free isolates. Yet, 54.5% of the CRISPR/Cas-positive isolates carried *bla*_NDM_. This contradicts the claims of a previous study that CRISPR/Cas significantly interferes with the acquisition of AmpC- and carbapenem-coding genes [29]. Contrary to our findings, a previous study conducted in China reported that the carbapenemase gene *bla*_NDM_ was more commonly carried by the CRISPR/Cas-positive isolates (40%) than the CRISPR/Cas-negative isolates (17%) [41]. Higher prevalence was also found for all ARGs among the I-E-positive isolates compared to those carrying I-E*, but with no statistical significance, except for *qnrS*. That contradicts a previous study by Li et al. that reported that the strains with type I-E* significantly harbored lower numbers of prophage regions, plasmids, and acquired ARGs [19].

Exploring the draft genomes of two representative isolates from our collection showed more spacers in I-E (50) than I-E* (21). More plasmids and more ARGs were also found in 117 that carried I-E* than in the I-E-positive isolate, K57. While this finding correlates well with the number of spacers in each isolate, it contradicts the findings of our PCR screening for CRISPR/Cas types and selected ARGs. Hence, a large-scale in silico analysis was conducted to better explore the correlation between CRISPR/Cas and resistance plasmids and ARGs.

The prevalence of the CRISPR/Cas system in 888 *K. pneumoniae* published genomes in silico-analyzed in the current study was comparable (23.2%) to the prevalence found in our collection. Type I-E* was also found in higher prevalence (53.8%) compared to I-E. The CRISPR-harboring genomes were widely distributed among unrelated STs, but the vast majority belonged to ST147, ST14, ST15, and the hypervirulent clone ST23, which is an important cause of carbapenem-resistant infections [52,53,54], with a reduction in carbapenemase resistance gene acquisition consistent with the former studies [18,19]. In agreement with others [27,55,56], none of the STs that belong to the high-risk clonal complex 258 that is responsible for about two-thirds of global human *K. pneumoniae* outbreaks [46,57] were found to carry CRISPR/Cas. Interestingly, 27/888 (3.0%) strains were found to harbor orphan CRISPR arrays and lack the *cas* gene cluster. As reported by Shen et al., this might be lost due to the existence of self-targeting spacers onboard [18]. Another previous study reported that more than half of self-targeting spacers are related to the loss of CRISPR’s own activity [58]. While we found solitary *cas3* genes on the plasmids carried by 8 out of 888 strains in silico-analyzed here, the PCR screening of the isolates collected in the current study showed that all *cas3*-positive strains also carried *cas1*. Similar findings were also reported by Wang et al. [29]. Within the analyzed genomes, we also found CRISPR arrays on 33 plasmids, including 17 plasmids that carried complete CRISPR/Cas loci on their genomes. Interestingly, most of them carried the replicon IncFIB. CRISPR-positive plasmids carried by *K. pneumoniae* were thoroughly studied by [20], who identified this type of CRISPR as type IV CRISPR/Cas that lacks *cas1* and *cas2* genes and suggested that these may acquire their competence from the chromosomal type I-E CRISPRs. The acquisition of CRISPR-positive plasmids is expected to augment the function of the chromosomal CRISPR/Cas, providing a wider spectrum of immunity against MGEs. Unexpectedly, this was associated with more resistance plasmids and ARGs. More analysis is required in this respect.

Plasmid counts and plasmid-encoded ARGs were apparently lower in CRISPR/Cas-bearing strains compared to strains lacking this system, consistent with a study by Li et al., but with no statistical significance. Consistent with the PCR findings, more plasmids and ARGs were carried by I-E strains compared to I-E*, but the difference in their distribution across the two groups was not statistically significant [19]. Our findings show that the CRISPR/Cas systems in *K. pneumoniae* are not always associated with a dearth of plasmid-borne ARGs, which is in good agreement with a recent study conducted in Egypt that reported the co-existence of an enormous number of ARGs, phages, and CRISPR/Cas systems in the analyzed genome [59]. Conflicting results were found for the distribution of individual plasmid-encoded ARGs and -bearing CRISPR/Cas systems. The prevalence of some ARGs was significantly higher among the CRISPR/Cas-negative strains. These included *bla*_TEM_, *bla*_LAP-2_, *bla*_KPC_, *rmtB*, *catA3*, and *fosA*. Other genes were significantly more common in the presence of CRISPR/Cas. These included *bla*_NDM_, *bla*_VIM_, *armA*, *florR*, *ereA2*, *msrE*, *tet*(B), and *mcr-3*, while the distribution of all other ARGs among the two groups was not statistically significant. Zhou et al. have also reported a lower prevalence of the carbapenemase-coding gene *bla*_KPC_ among the CRISPR/Cas-positive strains [30]. Other studies also showed that harboring CRISPR/Cas systems interferes with the incursion and survival of *bla_KPC_*-IncF plasmids [28,30]. Another prior work demonstrated that eliminating the CRISPR/Cas cassette in carbapenem-susceptible strains boosted the transformation success of *bla_KPC_* plasmids [27].

Among the analyzed published genomes, we, surprisingly, found a weak, nonsignificant positive correlation between the number of CRISPR spacers and the number of resistance plasmids and ARGs carried by the CRISPR/Cas-positive strains. In contrast, among the two isolates sequenced here, more spacers were associated with fewer plasmids and ARGs. However, analysis of the spacer sequences of the two isolates showed that not more than 8.0% of the spacers matched plasmid sequences. Hence, our hypothesis is that the number of spacers does not truly reflect the immunity against resistance plasmids. Rather, these are only targeted by a minority of spacers.

The contradictions in the impact of the CRISP/Cas system on limiting the dissemination of ARGs and, consequently, antimicrobial resistance was also evident from studies conducted on other bacterial species. The CRISPR/Cas system was previously found to be significantly associated with the absence of ARGs and high drug susceptibility in *Enterococcus faecalis* [22,23,60] and *Pseudomonas aeruginosa* [24,61,62]. On the other hand, it was associated with more antimicrobial resistance in *Campylobacter jejuni* [13]. There are a lot of reasons explaining why the existence of the CRSPR-Cas systems on the bacterial genome does not always impede the dissemination of ARGs, starting from the adaptation stage. Point mutations and insertion sequence-mediated mutations in the adaptation genes (*cas1* and *cas2*) correlated with the spread of MDR strains, as reported before in *Shigella* species [26]. In addition, strong selective pressure for antibiotic resistance may result in CRISPR repression [63], and many CRISPR-harboring strains may be immunologically inactive owing to the existence of self-targeting spacers, which would be anticipated to induce an autoimmune response and host cell death [18,55,58]. On the other hand, phages expressing anti-CRISPR proteins (Acrs) may inactivate the CRISPR/Cas system, resulting in the dissemination of ARGs, as found in *P. aeruginosa* [64,65,66,67,68]. Since matched proto-spacser sequences are required for CRISPR scanning, point mutations in PAM or mismatches between spacer and invader DNA abolish CRISPR interference and significantly reduce the affinity of the cascade-crRNA complex to target DNA, and cleavage does not occur, even though CRISPR is present [69,70,71]. Because the leader sequence acts as a promotor to regulate the transcription process and is a preferred site for the insertion of additional spacers, spacer GC content and proximity to the leader sequence are essential for CRISPR interference activity [71,72]. Furthermore, plasmid attacks are not only controlled by the CRISPR/Cas system, but additional roles are shared by the R-M (restriction–modification) systems [73]. In addition to DNA-binding proteins, H-NS proteins bind to the promoter of the cas operon, resulting in a decrease in cas3 expression and, consequently, a loss of CRISPR/Cas immunity. Previous research has shown that inhibiting Cas3 expression in *K. pneumoniae* by stimulating the transcriptional repressor H-NS results in loss of CRISPR/Cas immunity and increases plasmid transformation, and imipenem treatment causes loss of CRISPR activity through induction of H-NS expression [72,74,75,76,77]. Taken together, these reasons raise the possibility of mobile genetic elements evading CRISPR immunity. Inspired from our finding that only a few spacers matched plasmid sequences, we suggest that the acquisition of plasmid-matched spacers may not widely occur spontaneously, as they may adversely affect cellular fitness, particularly under antimicrobial treatment pressure. Contrarily, the majority of spacers are more likely to target prophages and other chromosomal sequences for gene regulation purposes. While the current study is the first in Egypt to explore the prevalence of CRISPR/Cas in clinical isolates of *K. pneumoniae*, it has the limitation that the draft genomes of only two CRISPR/Cas-positive isolates were generated. Hence, WGS of more CRISPR/Cas-positive strains coupled with spacer match analysis is recommended.

## 5. Conclusions

In conclusion, our study revealed that the CRISPR/Cas system is sparsely disseminated in the genomes of *K. pneumoniae* and is confined to specific STs. No significant correlation could be established between CRISPR/Cas and susceptibility to antimicrobial agents. The same was found for the carriage of antimicrobial resistance plasmids and onboard ARGs that were also unrelated to CRISPR/Cas spacer counts. Although we do not dispute the capacity of the CRISPR/Cas system to resist plasmid acquisition, which was experimentally proven by previous studies, we suggest that the incorporation of plasmid-matched spacers in the native CRISPR arrays does not happen spontaneously. This complicates the possibility of judging the clinical usefulness of CRISPR/Cas-based antimicrobials by merely investigating the association between the native CRISPR/Cas and antimicrobial resistance in clinical isolates. Hence, large-scale bioinformatic analysis of the exact matches of CRISPR spacers might provide a clear image of the exact behavior of CRISPR/Cas towards resistance plasmids. Furthermore, future research should focus on the prevalence of anti-CRISPR proteins and/or other proteins carried by the resistance plasmids that might provide them with immunity against a fully functional plasmid-targeted CRISPR/Cas system.

## Figures and Tables

**Table 1:** Features of the CRISPR/Cas-positive clinical isolates.

Isolate No	ST	CRISPR/Cas Type	Total No of Spacers	Plasmid Replicons	ARGs Carried on Plasmids
K57	ST1999	I-E	50	IncFIB(K)	*bla_TEM_*
117	ST870	I-E*	21	IncFIB(K) Col440I Col440II IncFIA (HI1) IncFII(pKP91) repB(R1701)	*bla*_DHA-1_*—tet*(D)*—aph(6)-Id—dfrA7—aph(3’)-Ia*

## Data Availability

Data supporting the reported results are available in the main manuscript and in the Supplementary File. The draft genome of the I-E-positive isolate K57 is available in the GenBank database under the BioProject number PRJNA844012. The draft genome of the I-E*-positive isolate 117 generated in the current study was submitted to the GenBank database under the BioProject number PRJNA992508 and under the accession code SUB13646323.

## Supplementary Materials

The following supporting information can be downloaded at: https://www.mdpi.com/article/10.3390/microorganisms11081948/s1, Table S1: List of primers used for polymerase chain reaction. Table S2: Relationship between the CRISPR-Cas system and the antibiotic resistance profile in *K. pneumoniae* clinical isolates. Table S3: Features of CRISPR/Cas loci identified in K57 and 117. Table S4: Features of the CRISPR-positive plasmids. Table S5: Association between resistance genes and CRISPR/Cas systems in *K. pneumoniae* published genomes.

## References

[1] Medina-Aparicio L., Davila S., Rebollar-Flores J.E., Calva E., Hernandez-Lucas I. (2018). The CRISPR-Cas system in *Enterobacteriaceae*. Pathog. Dis..

[2] Ishino Y., Shinagawa H., Makino K., Amemura M., Nakata A. (1987). Nucleotide sequence of the iap gene, responsible for alkaline phosphatase isozyme conversion in *Escherichia coli*, and identification of the gene product. J. Bacteriol..

[3] Ishino Y., Krupovic M., Forterre P. (2018). History of CRISPR-Cas from Encounter with a Mysterious Repeated Sequence to Genome Editing Technology. J. Bacteriol..

[4] Rath D., Amlinger L., Rath A., Lundgren M. (2015). The CRISPR-Cas immune system: Biology, mechanisms and applications. Biochimie.

[5] Garneau J.E., Dupuis M.E., Villion M., Romero D.A., Barrangou R., Boyaval P., Fremaux C., Horvath P., Magadan A.H., Moineau S. (2010). The CRISPR/Cas bacterial immune system cleaves bacteriophage and plasmid DNA. Nature.

[6] Barrangou R. (2013). CRISPR-Cas systems and RNA-guided interference. Wiley Interdiscip. Rev. RNA.

[7] Makarova K.S., Koonin E.V. (2015). Annotation and Classification of CRISPR-Cas Systems. Methods Mol. Biol..

[8] Makarova K.S., Wolf Y.I., Iranzo J., Shmakov S.A., Alkhnbashi O.S., Brouns S.J.J., Charpentier E., Cheng D., Haft D.H., Horvath P. (2020). Evolutionary classification of CRISPR-Cas systems: A burst of class 2 and derived variants. Nat. Rev. Microbiol..

[9] Wu Y., Battalapalli D., Hakeem M.J., Selamneni V., Zhang P., Draz M.S., Ruan Z. (2021). Engineered CRISPR-Cas systems for the detection and control of antibiotic-resistant infections. J. Nanobiotechnol..

[10] Abdelaziz S.M., Aboshanab K.M., Yahia I.S., Yassien M.A., Hassouna N.A. (2021). Correlation between the Antibiotic Resistance Genes and Susceptibility to Antibiotics among the Carbapenem-Resistant Gram-Negative Pathogens. Antibiotics.

[11] Xu Z., Li M., Li Y., Cao H., Miao L., Xu Z., Higuchi Y., Yamasaki S., Nishino K., Woo P.C.Y. (2019). Native CRISPR-Cas-Mediated Genome Editing Enables Dissecting and Sensitizing Clinical Multidrug-Resistant *P. aeruginosa*. Cell Rep..

[12] Touchon M., Charpentier S., Pognard D., Picard B., Arlet G., Rocha E.P., Denamur E., Branger C. (2012). Antibiotic resistance plasmids spread among natural isolates of *Escherichia coli* in spite of CRISPR elements. Microbiology.

[13] Shabbir M.A., Wu Q., Shabbir M.Z., Sajid A., Ahmed S., Sattar A., Tang Y., Li J., Maan M.K., Hao H. (2018). The CRISPR-cas system promotes antimicrobial resistance in *Campylobacter jejuni*. Future Microbiol..

[14] McDonald N.D., Regmi A., Morreale D.P., Borowski J.D., Boyd E.F. (2019). CRISPR-Cas systems are present predominantly on mobile genetic elements in *Vibrio* species. BMC Genom..

[15] Mohd Asri N.A., Ahmad S., Mohamud R., Mohd Hanafi N., Mohd Zaidi N.F., Irekeola A.A., Shueb R.H., Yee L.C., Mohd Noor N., Mustafa F.H. (2021). Global Prevalence of Nosocomial Multidrug-Resistant Klebsiella pneumoniae: A Systematic Review and Meta-Analysis. Antibiotics.

[16] Wyres K.L., Lam M.M.C., Holt K.E. (2020). Population genomics of *Klebsiella pneumoniae*. Nat. Rev. Microbiol..

[17] Ostria-Hernandez M.L., Sanchez-Vallejo C.J., Ibarra J.A., Castro-Escarpulli G. (2015). Survey of clustered regularly interspaced short palindromic repeats and their associated Cas proteins (CRISPR/Cas) systems in multiple sequenced strains of *Klebsiella pneumoniae*. BMC Res. Notes.

[18] Shen J., Lv L., Wang X., Xiu Z., Chen G. (2017). Comparative analysis of CRISPR-Cas systems in *Klebsiella* genomes. J. Basic. Microbiol..

[19] Li H.Y., Kao C.Y., Lin W.H., Zheng P.X., Yan J.J., Wang M.C., Teng C.H., Tseng C.C., Wu J.J. (2018). Characterization of CRISPR-Cas Systems in Clinical *Klebsiella pneumoniae* Isolates Uncovers Its Potential Association with Antibiotic Susceptibility. Front. Microbiol..

[20] Kamruzzaman M., Iredell J.R. (2019). CRISPR-Cas System in Antibiotic Resistance Plasmids in *Klebsiella pneumoniae*. Front. Microbiol..

[21] Chen S., Liu H., Liang W., Hong L., Zhang B., Huang L., Guo X., Duan G. (2019). Insertion sequences in the CRISPR-Cas system regulate horizontal antimicrobial resistance gene transfer in *Shigella* strains. Int. J. Antimicrob. Agents.

[22] Gholizadeh P., Aghazadeh M., Ghotaslou R., Rezaee M.A., Pirzadeh T., Cui L., Watanabe S., Feizi H., Kadkhoda H., Kafil H.S. (2021). Role of CRISPR-Cas system on antibiotic resistance patterns of *Enterococcus faecalis*. Ann. Clin. Microbiol. Antimicrob..

[23] Alduhaidhawi A.H.M., AlHuchaimi S.N., Al-Mayah T.A., Al-Ouqaili M.T.S., Alkafaas S.S., Muthupandian S., Saki M. (2022). Prevalence of CRISPR-Cas Systems and Their Possible Association with Antibiotic Resistance in *Enterococcus faecalis* and *Enterococcus faecium* Collected from Hospital Wastewater. Infect. Drug Resist..

[24] Soliman M., Said H.S., El-Mowafy M., Barwa R. (2022). Novel PCR detection of CRISPR/Cas systems in *Pseudomonas aeruginosa* and its correlation with antibiotic resistance. Appl. Microbiol. Biotechnol..

[25] Aydin S., Personne Y., Newire E., Laverick R., Russell O., Roberts A.P., Enne V.I. (2017). Presence of Type I-F CRISPR/Cas systems is associated with antimicrobial susceptibility in *Escherichia coli*. J. Antimicrob. Chemother..

[26] Ren L., Deng L.H., Zhang R.P., Wang C.D., Li D.S., Xi L.X., Chen Z.R., Yang R., Huang J., Zeng Y.R. (2017). Relationship between drug resistance and the clustered, regularly interspaced, short, palindromic repeat-associated protein genes cas1 and cas2 in Shigella from giant panda dung. Medicine.

[27] Mackow N.A., Shen J., Adnan M., Khan A.S., Fries B.C., Diago-Navarro E. (2019). CRISPR-Cas influences the acquisition of antibiotic resistance in *Klebsiella pneumoniae*. PLoS ONE.

[28] Tang Y., Fu P., Zhou Y., Xie Y., Jin J., Wang B., Yu L., Huang Y., Li G., Li M. (2020). Absence of the type I-E CRISPR-Cas system in *Klebsiella pneumoniae* clonal complex 258 is associated with dissemination of IncF epidemic resistance plasmids in this clonal complex. J. Antimicrob. Chemother..

[29] Wang G., Song G., Xu Y. (2020). Association of CRISPR/Cas System with the Drug Resistance in *Klebsiella pneumoniae*. Infect. Drug Resist..

[30] Zhou Y., Tang Y., Fu P., Tian D., Yu L., Huang Y., Li G., Li M., Wang Y., Yang Z. (2020). The type I-E CRISPR-Cas system influences the acquisition of bla(KPC)-IncF plasmid in *Klebsiella pneumonia*. Emerg. Microbes Infect..

[31] Liu Y., Wan L.G., Deng Q., Cao X.W., Yu Y., Xu Q.F. (2015). First description of NDM-1-, KPC-2-, VIM-2- and IMP-4-producing *Klebsiella pneumoniae* strains in a single Chinese teaching hospital. Epidemiol. Infect..

[32] Endimiani A., Carias L.L., Hujer A.M., Bethel C.R., Hujer K.M., Perez F., Hutton R.A., Fox W.R., Hall G.S., Jacobs M.R. (2008). Presence of plasmid-mediated quinolone resistance in *Klebsiella pneumoniae* isolates possessing blaKPC in the United States. Antimicrob. Agents Chemother..

[33] Yu Y., Ji S., Chen Y., Zhou W., Wei Z., Li L., Ma Y. (2007). Resistance of strains producing extended-spectrum beta-lactamases and genotype distribution in China. J. Infect..

[34] EUCAST Breakpoint Tables for Interpretation of MICs and Zone Diameters. Version 13.0. http://www.eucast.org.

[35] Magiorakos A.P., Srinivasan A., Carey R.B., Carmeli Y., Falagas M.E., Giske C.G., Harbarth S., Hindler J.F., Kahlmeter G., Olsson-Liljequist B. (2012). Multidrug-resistant, extensively drug-resistant and pandrug-resistant bacteria: An international expert proposal for interim standard definitions for acquired resistance. Clin. Microbiol. Infect..

[36] Chen Y., Chen Y., Shi C., Huang Z., Zhang Y., Li S., Li Y., Ye J., Yu C., Li Z. (2018). SOAPnuke: A MapReduce acceleration-supported software for integrated quality control and preprocessing of high-throughput sequencing data. Gigascience.

[37] Tatusova T., DiCuccio M., Badretdin A., Chetvernin V., Nawrocki E.P., Zaslavsky L., Lomsadze A., Pruitt K.D., Borodovsky M., Ostell J. (2016). NCBI prokaryotic genome annotation pipeline. Nucleic Acids Res..

[38] Carattoli A., Zankari E., Garcia-Fernandez A., Voldby Larsen M., Lund O., Villa L., Moller Aarestrup F., Hasman H. (2014). In silico detection and typing of plasmids using PlasmidFinder and plasmid multilocus sequence typing. Antimicrob. Agents Chemother..

[39] Arredondo-Alonso S., Rogers M.R.C., Braat J.C., Verschuuren T.D., Top J., Corander J., Willems R.J.L., Schurch A.C. (2018). mlplasmids: A user-friendly tool to predict plasmid- and chromosome-derived sequences for single species. Microb. Genom..

[40] Hu Y., Jiang J., Wang D., Guo Q., Wang M. (2023). Coexistence of bla (KPC)-IncFII plasmids and type I-E(*) CRISPR-Cas systems in ST15 *Klebsiella pneumoniae*. Front. Microbiol..

[41] Liao W., Liu Y., Chen C., Li J., Du F., Long D., Zhang W. (2020). Distribution of CRISPR-Cas Systems in Clinical Carbapenem-Resistant Klebsiella pneumoniae Strains in a Chinese Tertiary Hospital and Its Potential Relationship with Virulence. Microb. Drug Resist..

[42] Sinkunas T., Gasiunas G., Fremaux C., Barrangou R., Horvath P., Siksnys V. (2011). Cas3 is a single-stranded DNA nuclease and ATP-dependent helicase in the CRISPR/Cas immune system. EMBO J..

[43] Farhadi M., Ahanjan M., Goli H.R., Haghshenas M.R., Gholami M. (2021). High frequency of multidrug-resistant (MDR) *Klebsiella pneumoniae* harboring several beta-lactamase and integron genes collected from several hospitals in the north of Iran. Ann. Clin. Microbiol. Antimicrob..

[44] Nakamura-Silva R., Cerdeira L., Oliveira-Silva M., da Costa K.R.C., Sano E., Fuga B., Moura Q., Esposito F., Lincopan N., Wyres K. (2022). Multidrug-resistant *Klebsiella pneumoniae*: A retrospective study in Manaus, Brazil. Arch. Microbiol..

[45] Fursova N.K., Astashkin E.I., Ershova O.N., Aleksandrova I.A., Savin I.A., Novikova T.S., Fedyukina G.N., Kislichkina A.A., Fursov M.V., Kuzina E.S. (2021). Multidrug-Resistant *Klebsiella pneumoniae* Causing Severe Infections in the Neuro-ICU. Antibiotics.

[46] Navon-Venezia S., Kondratyeva K., Carattoli A. (2017). Klebsiella pneumoniae: A major worldwide source and shuttle for antibiotic resistance. FEMS Microbiol. Rev..

[47] Mostafa S.H., Saleh S.E., Hamed S.M., Aboshanab K.M. (2022). Febrile illness of bacterial etiology in a public fever hospital in Egypt: High burden of multidrug resistance and WHO priority Gram negative pathogens. Germs.

[48] Hassuna N.A., AbdelAziz R.A., Zakaria A., Abdelhakeem M. (2020). Extensively-Drug Resistant *Klebsiella pneumoniae* Recovered From Neonatal Sepsis Cases From a Major NICU in Egypt. Front. Microbiol..

[49] El-Badawy M.F., Tawakol W.M., El-Far S.W., Maghrabi I.A., Al-Ghamdi S.A., Mansy M.S., Ashour M.S., Shohayeb M.M. (2017). Molecular Identification of Aminoglycoside-Modifying Enzymes and Plasmid-Mediated Quinolone Resistance Genes among *Klebsiella pneumoniae* Clinical Isolates Recovered from Egyptian Patients. Int. J. Microbiol..

[50] Hamed S.M., Aboshanab K.M.A., El-Mahallawy H.A., Helmy M.M., Ashour M.S., Elkhatib W.F. (2018). Plasmid-Mediated Quinolone Resistance in Gram-Negative Pathogens Isolated from Cancer Patients in Egypt. Microb. Drug Resist..

[51] Jlili N.E., Rejiba S., Smaoui H., Guillard T., Chau F., Kechrid A., Cambau E. (2014). Trend of plasmid-mediated quinolone resistance genes at the Children’s Hospital in Tunisia. J. Med. Microbiol..

[52] Kumar V., Sun P., Vamathevan J., Li Y., Ingraham K., Palmer L., Huang J., Brown J.R. (2011). Comparative genomics of Klebsiella pneumoniae strains with different antibiotic resistance profiles. Antimicrob. Agents Chemother..

[53] Bolourchi N., Naz A., Sohrabi M., Badmasti F. (2022). Comparative in silico characterization of Klebsiella pneumoniae hypervirulent plasmids and their antimicrobial resistance genes. Ann. Clin. Microbiol. Antimicrob..

[54] David S., Reuter S., Harris S.R., Glasner C., Feltwell T., Argimon S., Abudahab K., Goater R., Giani T., Errico G. (2019). Epidemic of carbapenem-resistant Klebsiella pneumoniae in Europe is driven by nosocomial spread. Nat. Microbiol..

[55] Shen J., Zhou J., Xu Y., Xiu Z. (2020). Prophages contribute to genome plasticity of *Klebsiella pneumoniae* and may involve the chromosomal integration of ARGs in CG258. Genomics.

[56] Huang W., Wang G., Sebra R., Zhuge J., Yin C., Aguero-Rosenfeld M.E., Schuetz A.N., Dimitrova N., Fallon J.T. (2017). Emergence and Evolution of Multidrug-Resistant *Klebsiella pneumoniae* with both bla(KPC) and bla(CTX-M) Integrated in the Chromosome. Antimicrob. Agents Chemother..

[57] Bialek-Davenet S., Criscuolo A., Ailloud F., Passet V., Jones L., Delannoy-Vieillard A.S., Garin B., Le Hello S., Arlet G., Nicolas-Chanoine M.H. (2014). Genomic definition of hypervirulent and multidrug-resistant *Klebsiella pneumoniae* clonal groups. Emerg. Infect. Dis..

[58] Stern A., Keren L., Wurtzel O., Amitai G., Sorek R. (2010). Self-targeting by CRISPR: Gene regulation or autoimmunity?. Trends Genet..

[59] Enany S., Zakeer S., Diab A.A., Bakry U., Sayed A.A. (2022). Whole genome sequencing of Klebsiella pneumoniae clinical isolates sequence type 627 isolated from Egyptian patients. PLoS ONE.

[60] Palmer K.L., Gilmore M.S. (2010). Multidrug-resistant enterococci lack CRISPR-cas. mBio.

[61] van Belkum A., Soriaga L.B., LaFave M.C., Akella S., Veyrieras J.B., Barbu E.M., Shortridge D., Blanc B., Hannum G., Zambardi G. (2015). Phylogenetic Distribution of CRISPR-Cas Systems in Antibiotic-Resistant *Pseudomonas aeruginosa*. mBio.

[62] Hatoum-Aslan A., Marraffini L.A. (2014). Impact of CRISPR immunity on the emergence and virulence of bacterial pathogens. Curr. Opin. Microbiol..

[63] Bikard D., Hatoum-Aslan A., Mucida D., Marraffini L.A. (2012). CRISPR interference can prevent natural transformation and virulence acquisition during in vivo bacterial infection. Cell Host Microbe.

[64] Sontheimer E.J., Davidson A.R. (2017). Inhibition of CRISPR-Cas systems by mobile genetic elements. Curr. Opin. Microbiol..

[65] Marino N.D., Pinilla-Redondo R., Csorgo B., Bondy-Denomy J. (2020). Anti-CRISPR protein applications: Natural brakes for CRISPR-Cas technologies. Nat. Methods.

[66] Davidson A.R., Lu W.T., Stanley S.Y., Wang J., Mejdani M., Trost C.N., Hicks B.T., Lee J., Sontheimer E.J. (2020). Anti-CRISPRs: Protein Inhibitors of CRISPR-Cas Systems. Annu. Rev. Biochem..

[67] Bondy-Denomy J., Pawluk A., Maxwell K.L., Davidson A.R. (2013). Bacteriophage genes that inactivate the CRISPR/Cas bacterial immune system. Nature.

[68] Pawluk A., Bondy-Denomy J., Cheung V.H., Maxwell K.L., Davidson A.R. (2014). A new group of phage anti-CRISPR genes inhibits the type I-E CRISPR-Cas system of *Pseudomonas aeruginosa*. mBio.

[69] Watson B.N.J., Steens J.A., Staals R.H.J., Westra E.R., van Houte S. (2021). Coevolution between bacterial CRISPR-Cas systems and their bacteriophages. Cell Host Microbe.

[70] Westra E.R., Semenova E., Datsenko K.A., Jackson R.N., Wiedenheft B., Severinov K., Brouns S.J. (2013). Type I-E CRISPR-cas systems discriminate target from non-target DNA through base pairing-independent PAM recognition. PLoS Genet..

[71] Fu B.X., Wainberg M., Kundaje A., Fire A.Z. (2017). High-Throughput Characterization of Cascade type I-E CRISPR Guide Efficacy Reveals Unexpected PAM Diversity and Target Sequence Preferences. Genetics.

[72] Pul U., Wurm R., Arslan Z., Geissen R., Hofmann N., Wagner R. (2010). Identification and characterization of *E. coli* CRISPR-cas promoters and their silencing by H-NS. Mol. Microbiol..

[73] Wyres K.L., Wick R.R., Judd L.M., Froumine R., Tokolyi A., Gorrie C.L., Lam M.M.C., Duchene S., Jenney A., Holt K.E. (2019). Distinct evolutionary dynamics of horizontal gene transfer in drug resistant and virulent clones of *Klebsiella pneumoniae*. PLoS Genet..

[74] Lin T.L., Pan Y.J., Hsieh P.F., Hsu C.R., Wu M.C., Wang J.T. (2016). Imipenem represses CRISPR-Cas interference of DNA acquisition through H-NS stimulation in *Klebsiella pneumoniae*. Sci. Rep..

[75] Westra E.R., Pul U., Heidrich N., Jore M.M., Lundgren M., Stratmann T., Wurm R., Raine A., Mescher M., Van Heereveld L. (2010). H-NS-mediated repression of CRISPR-based immunity in *Escherichia coli* K12 can be relieved by the transcription activator LeuO. Mol. Microbiol..

[76] Majsec K., Bolt E.L., Ivancic-Bace I. (2016). Cas3 is a limiting factor for CRISPR-Cas immunity in *Escherichia coli* cells lacking H-NS. BMC Microbiol..

[77] Stoebel D.M., Free A., Dorman C.J. (2008). Anti-silencing: Overcoming H-NS-mediated repression of transcription in Gram-negative enteric bacteria. Microbiology.

