# Supplementary material for: Correlation of CRISPR/Cas and Antimicrobial Resistance in Klebsiella pneumoniae Clinical Isolates Recovered from Patients in Egypt Compared to Global Strains
Source: Microorganisms. 2023 Jul 30;11(8):1948. doi: 10.3390/microorganisms11081948 (PMC10459600; doi:10.3390/microorganisms11081948)
Supplement: Supplementary file 1 [file microorganisms-11-01948-s001.zip › microorganisms-2536753-supplementary.pdf]

Table S1: List of primers used for polymerase chain reaction

Gene	Primer	Sequence (5`-3`)	T_a	Product (bp)	Reference
cas1 form A (I-E)	Cas1A-F	CGAAACGCTACGGTGTGAAA	60 °C	341	(Li et al., 2018)
	Cas1A-R	CGGAATCAATTTGCCTGTCA			
cas1 form B (I-E*)	Cas1B-F	CGGCTGGAAATTGATGACAG		309	
	Cas1B-R	ATCCGGAAAGCGTTTAGCAA			
cas3 form A (I-E)	Cas3A-F	GCCGCTTTGCCTTTTATGAC	66 °C	362	
	Cas3A-R	GCCGTGATCGGGATAGACAT			
cas3 form B (I-E*)	Cas3B-F	AGTCTGGAGGTCTGGCAAGC		407	
	Cas3B-F	CGCACCAGCTGCTGATTAAG			
acc (6`)-lb	*aac(6`)-Ib-F*	CTTGCGATGCTCTATGAGTGG	60 °C	480	(Hamed et al., 2018)
	aac(6`)-Ib-R	GAATGCCTGGCGTGTTTGAA			
qnrS	*qnrS-F*	TCGGCACCACAACTTTTCAC		255	
	qnrS-R	TCACACGCACGGAACTCTAT			
qnrB	*qnrB-F*	TATGGCTCTGGCACTCGTT		193	
	qnrB-R	GCATCTTTCAGCATCGCAC			
bla_{CTX-M}	*CTX-M-F*	TTTGCGATGTGCAGTACCAGTAA	47 °C	544	(Edelstein et al., 2003)
	CTX-M-R	CGATATCGTTGGTGGTGCCATA			
bla_{SHV}	*SHV-F*	AAGATCCACTATCGCCAGCAG	64 °C	234	(Poirel et al., 2004)
	SHV-R	ATTCAGTTCCGTTTCCCAGCGG			
bla_{NDM}	*NDM-F*	GGTTTGGCGATCTGGTTTTC	53 °C	621	(Poirel et al., 2011)
	NDM-R	CGGAATGGCTCATCACGATC			
armA	*armA-F*	TATGGGGGTCTTACTATTCTGCCTAT	58 °C	518	(Fritsche et al., 2008)
	armA-R	TCTTCCATTCCCTTCTCCTTT			

Ta, annealing temperature.

Table S2: Relationship between the CRISPR-Cas system and the antibiotic resistance profile in *K. pneumoniae* clinical isolates

**Antimicrobial agents**	**No. of resistant isolates**		**P-value***
	**Cas-positive (n=46)**	**Cas-negative (n=135)**	
Amoxicillin/Clavulanic acid	45 (97.8%)	135 (100%)	0.066
Cefepime	46 (100%)	135 (100%)	NA
Cefotaxime	44 (95.6%)	129 (95.6%)	0.697
Ceftriaxone	44 (95.6%)	128 (94.8%)	0.720
Cefoxitin	38 (82.6%)	116 (86%)	0.406
Ceftazidime	44 (95.6%)	132 (97.8%)	0.179
Aztreonam	42 (91.3%)	127 (94.1%)	0.325
Imipenem	35 (76.1%)	110 (81.5%)	0.304
Gentamicin	38 (82.6%)	93 (68.9%)	0.116
Amikacin	32 (69.6%)	95 (70.4%)	0.769
Tetracycline	28 (60.9%)	72 (53.3%)	0.459
Ciprofloxacin	41 (89.1%)	127 (94.1%)	0.199
Trimethoprim-Sulfamethoxazole	40 (87%)	120 (88.9%)	0.493
Chloramphenicol	30 (65.2%)	76 (56.3%)	0.367
Nitrofurantoin	41(89.1%)	126 (93.3%)	0.220
Tigecycline	8 (17.4%)	19 (14%)	0.624
Colistin	3 (6.5%)	1 (0.7%)	0.054

*P-values were calculated by Chi Square or Fisher's Exact tests where appropriate

Table S3: Features of CRISPR/Cas loci identified in K57 and 117

**Features**	**K57**	**117**
No. of CRISPR arrays	1	2
Repeat Consensus:	gtgttccccgcgccagcggggataaaccg	gaaacacccccacgtgcgtggggaagac<br>gaaacacccccacgcatgtggggaagac
Repeat Length:	29 bp	28 bp
Total No. of Spacers	50	21
Mean size Spacers:	31.94 bp	33 bp
Spacers sequences:	>spacer1<br>gcgatcatgccgtagtcttcgatcacgtaatc<br>>spacer2<br>tttgccttcctgtctgcagaggcgcggacagg<br>>spacer3<br>cgtagctaaaattaactgcattcggcgcacct<br>>spacer4<br>ccagctcaatttccgcctgtcccgccagcgcc<br>>spacer5<br>aatgcagcaaccggcaaatatatcgccggtaa<br>>spacer6<br>ccgcaataacaaaaataaatgagggttaaagt<br>>spacer7<br>gtaaatgggaatgagtagaagagcgtcattg<br>g<br>>spacer8<br>attaaatttttcgggtaaagagaaattgcgaa<br>>spacer9<br>ctgcgagctaccctgaatttcagcgacagggg<br>>spacer10<br>tagtgccatatattatcctctgcggtatgact<br>>spacer11<br>gagactattatgcgaaaaactattactcccga<br>>spacer12<br>cccccgcgcacatgcttaaacgcgctatcacg<br>>spacer13<br>ggcatctgttgtgtaatgttgagttttttca<br>>spacer14<br>caggttaaacatgtaaaaaatgaccgtcgccg<br>>spacer15<br>cacattgcccggtctgaaaagtatttgaaaat<br>>spacer16<br>ccggaacaccaccagtaacagctactgtaggc<br>>spacer17<br>tgaccctgttgattttgttacaggtaatacgt<br>>spacer18<br>ttaacctcgtcgttctggtttccgcccaggat<br>>spacer19<br>gaacctgaattcgaagggtgggtcatccttcc<br>>spacer20<br>ttgagtgacagagagattgaccgccttgtttt<br>>spacer21	<u>**CRISPR1:**</u><br>>spacer1<br>agaacgaatgcccgcgctggtacggcgcgtcg<br>tggattcca<br>>spacer2<br>tgccggatatcatcaccgcgattaaacggcgga<br>>spacer3<br>cacacggcggcgtcgttgggccactccgaaata<br>>spacer4<br>tcttgccgatattgatatacacgccttttgcga<br>>spacer5<br>actcactcaatagtgtctctatctccgccatgg<br>>spacer6<br>cgacacaatgagccgcctccgggcggactattg<br>>spacer7<br>gcggtgaaccttggggcgtctcctcgcgaacta<br>>spacer8<br>gctaaccagtggatagagcactatgtgacgaca<br>>spacer9<br>gcccagggccgcgacgcggtccaggtgctcct<br>g<br>>spacer10<br>cggcagcagaacggcatgatgacgcggatttt<br>a<br>>spacer11<br>aataaccccggctttgaagtcattcagcccata<br>>spacer12<br>ccagacaaagctttgcgtcggcgaacgacgac<br>a<br>>spacer13<br>caggcttttaactgatccgcatagatcacctga<br>>spacer14<br>ctccccgtctttcggctccggccactcgcgttg<br><br><u>**CRISPR2:**</u><br>>spacer1<br>aggatagagtcaaatccgctcacacgtgatgaa<br>>spacer2<br>ccggcatccgtcagctcgacggccagctgcag<br>g<br>>spacer3<br>acccgcagcgctatcccgccgcagatgatggaa

Features	K57	117
	gtcagatcacgtcaaccacgcttgattttacc<br>>spacer22<br>ctcactaacgccgttgcctgcgatagctgatg<br>>spacer23<br>gtgatcggcccggcaatgcgtgagctaatcaa<br>>spacer24<br>ttgagtgacagagagattgaccgccttgtttt<br>>spacer25<br>aactgaatgttccaaatccggttggacgacca<br>>spacer26<br>ttcagcagcgcgctcgtccccctcctgtcgga<br>>spacer27<br>ctctactacgataacggatcccacgcgggctg<br>>spacer28<br>gctgccagcccattccgtaattgcctgtttca<br>>spacer29<br>gcgaatataggcattaatgcttttaataatat<br>>spacer30<br>ctccgtcaccagttctgtgctatccggcacaa<br>>spacer31<br>tcacgtttgcagaactcgcggaggaagaaga<br>a<br>>spacer32<br>gatcccccggaggagactgatcaaaccactc<br>g<br>>spacer33<br>cgctggttaatgggcttcccggaagggtggg<br>c<br>>spacer34<br>tctatcgcaaccaccgctgaggcgctgattga<br>>spacer35<br>ccgctccacggtgagcggctgttcaatgtagt<br>>spacer36<br>catcacgcgctggccaatgtgttaaaccaaat<br>>spacer37<br>ggcatcattctcttcgctactcccgccgatca<br>>spacer38<br>cggaaattttgattagttgaatctgtgccatt<br>>spacer39<br>ccgacttgggacgaggatccggcggaatgtc<br>g<br>>spacer40<br>taatggcaaaaccatgacctgatccgggcgtc<br>>spacer41<br>cctgaatcttcacttcgtcgatcattctgcgc<br>>spacer42<br>ggtcaaaaggttcattcgagcatcgagttgca<br>>spacer43<br>gtaacgcagacggcgaacgtcggatccattg<br>g	>spacer4<br>ccgcatgcggaagtcgaaataccacaccgacg<br>a<br>>spacer5<br>tcaatgaggctatgagagaggttgaggcgctc<br>a<br>>spacer6<br>tgtccccacttggcgccggggtaaagttgtcag<br>>spacer7<br>actattcgcaaaaatccggttttaccggcgtta

Features	K57	117
	>spacer44<br>gaccagaaagcctgggcattgttccgctcatt<br>>spacer45<br>cccccggccgcgtggccgattgccattaccgc<br>>spacer46<br>ttaataccagggggcaggttcagcaggtcccc<br>>spacer47<br>ccgctttaacccgctccggcagatccgggtga<br>>spacer48<br>cgcgctgcgaatttgttggtcgatttcgatct<br>>spacer49<br>cagacagacagcaggcagcaaacagggaa<br>gac<br>>spacer50<br>gggttcacttgggtgaaactgaactaact	

Spacers written in red match plasmid sequences, while spacers written in blue have no similarity in the nt/nr NCBI database. Other spacers match chromosomal sequences.

Table S4: Features of the CRISPR-positive plasmids

Strain	Plasmid-Accession No	Replicon type	Antibiotic resistance genes (By R.G.I)	No of Spacers
PittNDM01	NZ_CP006799.1	IncHI1B, IncFIB	*aac (6')-Ib-cr6, bla*NDM-1, *aph (3')-VI,mphE,msrE, armA,sul1,qacEdelta1,aadA2,dfrA12,dfrA14,bla*OXA1*,qnrB*	12
KP617	NZ_CP012754.1	IncHI1B, IncFIB	*mphE, msrE, armA, sul1, qacEdelta1, aadA2, dfrA12, qnrB1, bla*NDM-1, *aph (3')-VI*	12
11	NZ_CP016921.1	IncHI1B, IncFIB	*qnrB1, bla*OXA1, *dfrA14, dfrA12, aadA2, qacEdelta1, sul1, armA, msrE,mphE,aph(3')-VI,bla*NDM-1,*acc(6')-Ib-cr6*	12
825795-1	NZ_CP017986.1	IncHI1B, IncFIB	None	17
AR_0068	NZ_CP020068.1	IncHI1B, IncFIB	*dfrA12, aadA2, qacEdelta1, sul1, armA, msrE, mphE, aph (3')-VI, bla*NDM-1, *aac (3)-Iid, bla*SHV-11, *sul2, aph(6)-Id,aph(3'')-Ib*	12
KPN528	NZ_CP020854.1	IncHI1B, IncFIB	*qnrB1, bla*OXA-1, *dfrA12, aadA2, qacEdelta1, sul1, armA, msrE,mphE,aph(3')-VI, bla*NDM-1,*aac(6')-Ib-cr6,dfrA14*	12
AR_0153	NZ_CP028929.1	IncHI1B, IncFIB	*dfrA12, aadA2, qacEdelta1, sul1, armA, msrE, mphE,aph(3')-VI,bla*NDM-1,*qnrB1,bla*OXA-1,*dfrA14,aac(6')- Ib-cr6*	12
CDC 0106	NZ_CP022612.1	IncFIA, IncR, IncFIB, IncHI1B	*sul2, cmlA5, arr-2, bla-*SHV-11, *aac (3)-Iid, bla-*CTX-M-15, *bla-*OXA-9, *bla*TEM-1, *bla*NDM-1, *aph (3')-VI, mphE, msrE, armA, sul1, qacEdelta1, aadA2, dfrA12, aph (3'')-Ib,aph (6)-Id,sul1,ereA2,aac(6')-Ib10,aadA*	12
KP33	NZ_AP018748.1	IncHI1B, IncFIB	*bla*OXA-1, *qnrB1, dfrA14, tet(D), bla*TEM-1, *bla*CTX-M-15, *dfrA12, aadA2, qacEdelta1, sul1, armA, msrE, mphE, aph(3')-VI,aac(6')-Ib-cr6,aac(6')-Ib10, bla*NDM-28	11
M5	CP031735.1	IncFIB(K), IncFII(K)	*bla*TEM-1, *bla*CTX-M-15, *catI, dfrA14, aac (6')-Ib10*	9
AR_0046	NZ_CP032223.1	IncHI1B, IncFIB	None	13
NH34	NZ_CP034406.1	IncHI1B, IncFIB	*bla*OXA-1, *aph (3')-VI, mphE, msrE, armA, sul1, qacEdelta1, aadA2, dfrA12, bla*CTX-M-15, *bla*TEM-1, *qnrB1, aac (6')-Ib10, tet(D), dfrA14, aac (6')-Ib-cr6*	12
BP327	NZ_CP036336.1	IncHI1B	*sul2, bla*TEM-1, *bla*CTX-M-15, *dfrA12, qacEdelta1, sul1, armA,msrE,mphE,aph(3'')-Ib,aph(6)-Id,aadA2*	7
2018S07-013	CP044381.1	IncHI1B, IncFIB, IncN	*sul1, bla*TEM-1, *sul3, bla*DHA-1, *qnrB4, aac(3)-Iic,aph(3'')-Ib,aph(6)-Id,qacl,aadA2*	22
BP3636	NZ_CP053772.1	IncHI1B, IncFIB	*dfrA12, aadA2, qacEdelta1, sul1, armA, msrE,mphE, dfrA14, tet(D), bla*TEM-1, *bla*CTX-M-15, *aac (6')-Ib-cr6, bla*OXA-1	12
IR5065	NZ_CP061949.1	IncHI1B, IncFIB	*aph (3')-Ia, sul1, tetR, qacEdelta1, ant(3'')-Iia,tet(B)*	19

COL17	NZ_CP072906.1	IncHI1B, IncFIB	*catI*, *bla*$_{OXA-1}$, *catB3*, *arr-3*, *qacEdelta1*, *sul1*, *bla*$_{NDM-1}$, *bla*$_{TEM-26}$, *qnrE2*, *aac (6')-Ib-cr6*, *aac (3)-Iie*, *adeF*	14

Table S5: Association between resistance genes and CRISPR/Cas systems in *K. pneumoniae* published genomes

Gene	Affected antimicrobial class	Resistance mechanism	CRISPR/Cas-positive (n=206)	CRISPR/Cas-negative (n= 682)	P-value
aac(6')-Ib /aac(3)	Aminoglycosides	Antibiotic inactivation	78 (37.9%)	254 (37.2%)	0.872
acc(6')-Ib-cr6	Aminoglycosides and fluoroquinolones	Antibiotic inactivation	51 (24.8%)	144 (21.1%)	0.268
aph(6)-Id	Aminoglycosides	Antibiotic inactivation	77 (37.4%)	267 (39.1%)	0.647
ant(3'')	Aminoglycosides	Antibiotic inactivation	11 (5.3%)	38 (5.6%)	0.898
bla_{CTX-M}	B-lactams	Antibiotic inactivation	81 (39.3%)	291 (42.7%)	0.393
bla_{DHA-1}	B-lactams	Antibiotic inactivation	5 (2.4%)	35 (5.1%)	0.101
bla_{CMY-4}	B-lactams	Antibiotic inactivation	8 (3.9%)	15 (2.2%)	0.182
bla_{SHV}	B-lactams	Antibiotic inactivation	24 (11.7%)	103 (15.1%)	0.215
bla_{OXA}	B-lactams	Antibiotic inactivation	57 (27.7%)	175 (25.7%)	0.565
bla_{NDM}	B-lactams	Antibiotic inactivation	40 (19.4%)	45 (6.6%)	0.000
bla_{VIM}	B-lactams	Antibiotic inactivation	6 (2.9%)	5 (0.7%)	0.023
bla_{TEM}	B-lactams	Antibiotic inactivation	74 (35.9%)	360 (52.8%)	0.000
bla_{LAP-2}	B-lactams	Antibiotic inactivation	2 (1%)	59 (8.7%)	0.000
bla_{KPC}	B-lactams	Antibiotic inactivation	18 (8.7%)	213 (31.2%)	0.000
bla_{FOX-5}	B-lactams	Antibiotic inactivation	1 (0.5%)	0 (0%)	0.232
bla_{FONA-6}	B-lactams	Antibiotic inactivation	1 (0.5%)	1 (0.1%)	0.410
bla_{IMP}	B-lactams	Antibiotic inactivation	2 (1%)	9 (1.3%)	1.000
bla_{VEB}	B-lactams	Antibiotic inactivation	2 (1%)	4 (0.6%)	0.627
bla_{SCO-1}	B-lactams	Antibiotic inactivation	0 (0%)	1 (0.1%)	1.000
armA	Aminoglycosides	Antibiotic target alteration	23 (11.2%)	23 (3.4%)	0.000
rmtB	Aminoglycosides	Antibiotic target alteration	5 (2.4%)	98 (14.4%)	0.000

Gene	Affected antimicrobial class	Resistance mechanism	CRISPR/Cas-positive (n=206)	CRISPR/Cas-negative (n= 682)	P-value
rmtC	Aminoglycosides	Antibiotic target alteration	2 (1%)	5 (0.7%)	0.666
rmtF	Aminoglycosides	Antibiotic target alteration	5 (2.4%)	21 (3.1%)	0.627
rmtG	Aminoglycosides	Antibiotic target alteration	0 (0%)	2 (0.3%)	1.000
floR	Phenicol antibiotic	Antibiotic efflux	9 (4.4%)	12 (1.8%)	0.038
ereA2	Macrolides	Antibiotic inactivation	8 (3.9%)	3 (0.4%)	0.001
msrE	Macrolides	Antibiotic target protection	26 (12.6%)	27 (4%)	0.000
catA3	Phenicol antibiotic	Antibiotic inactivation	35 (17%)	189 (27.7%)	0.002
fosA	Phosphonic acid antibiotic	Antibiotic inactivation	1 (0.5%)	44 (6.5%)	0.001
ICR-Mo	Peptide antibiotic	Antibiotic target alteration	0 (0%)	1 (0.1%)	1.000
oqxA	Fluoroquinolone antibiotic, glycylcycline	Antibiotic efflux	0 (0%)	1 (0.1%)	1.000
aadA	Aminoglycoside antibiotic	Antibiotic inactivation	55 (26.7%)	207 (30.4%)	0.314
tet(A)	Tetracycline antibiotic	Antibiotic efflux	46 (22.3%)	189 (27.7)	0.125
tet(B)	Tetracycline antibiotic	Antibiotic efflux	4 (1.9%)	2 (0.3%)	0.028
tet(D)	Tetracycline antibiotic	Antibiotic efflux	18 (8.7%)	63 (9.2%)	0.827
cmlA	Phenicol antibiotic	Antibiotic efflux	11 (5.3%)	51 (7.5%)	0.291
mphE	Macrolide antibiotic	Antibiotic inactivation	59 (28.6%)	188 (27.6%)	0.763
mefB	Macrolide antibiotic	Antibiotic efflux	1 (0.5%)	7 (1%)	0.689
sat-2	Nucleoside antibiotic	Antibiotic inactivation	1 (0.5%)	3 (0.4%)	1.000
mcr-1	Peptide antibiotic	Antibiotic target alteration	0 (0%)	5 (0.7%)	0.595

Gene	Affected antimicrobial class	Resistance mechanism	CRISPR/Cas-positive (n=206)	CRISPR/Cas-negative (n= 682)	P-value
mcr-3	Peptide antibiotic	Antibiotic target alteration	3 (1.5%)	1 (0.1%)	0.041
mcr-3.5	Peptide antibiotic	Antibiotic target alteration	1 (0.5%)	2 (0.3%)	0.547
mcr-8	Peptide antibiotic	Antibiotic target alteration	2 (1%)	9 (1.3%)	0.692
mcr-9	Peptide antibiotic	Antibiotic target alteration	0 (0%)	1 (0.1%)	1.000
dfrA	Diaminopyrimidine antibiotic	Antibiotic target replacement	89 (43.2%)	308 (45.2%)	0.620
qnrA	Fluoroquinolone antibiotic	Antibiotic target protection	1 (0.5%)	9 (1.3%)	0.320
qnrB	Fluoroquinolone antibiotic	Antibiotic target protection	40 (19.4%)	133 (19.5%)	0.979
qnrE	Fluoroquinolone antibiotic	Antibiotic target protection	1 (0.5%)	1(0.1%)	0.410
qnrS	Fluoroquinolone antibiotic	Antibiotic target protection	27 (13.1%)	99 (14.5%)	0.611
qacl	Disinfecting agents and antiseptics	Antibiotic efflux	3 (1.5%)	26 (3.8%)	0.095
qacEdelta	Disinfecting agents and antiseptics	Antibiotic efflux	68 (33%)	205 (30.1%)	0.421
sul	Sulfonamide antibiotic	Antibiotic target replacement	105 (51%)	371 (54.4%)	0.387
erm	Macrolides	Antibiotic efflux	9 (4.4%)	21(3.1%)	0369

Statistically significant P-values are written in red.

References:

Edelstein, M., Pimkin, M., Palagin, I., Edelstein, I., and Stratchounski, L. (2003). Prevalence and molecular epidemiology of CTX-M extended-spectrum beta-lactamase-producing Escherichia coli and Klebsiella pneumoniae in Russian hospitals. *Antimicrob Agents Chemother* 47**,** 3724-3732. doi: https://doi.org/10.1128/AAC.47.12.3724-3732.2003.

Fritsche, T.R., Castanheira, M., Miller, G.H., Jones, R.N., and Armstrong, E.S. (2008). Detection of methyltransferases conferring high-level resistance to aminoglycosides in enterobacteriaceae from Europe, North America, and Latin America. *Antimicrob Agents Chemother* 52**,** 1843-1845. doi: https://doi.org/10.1128/AAC.01477-07.

Hamed, S.M., Aboshanab, K.M.A., El-Mahallawy, H.A., Helmy, M.M., Ashour, M.S., and Elkhatib, W.F. (2018). Plasmid-Mediated Quinolone Resistance in Gram-Negative Pathogens Isolated from Cancer Patients in Egypt. *Microb Drug Resist* 24**,** 1316-1325. doi: https://doi.org/10.1089/mdr.2017.0354.

Li, H.Y., Kao, C.Y., Lin, W.H., Zheng, P.X., Yan, J.J., Wang, M.C., et al. (2018). Characterization of CRISPR-Cas Systems in Clinical Klebsiella pneumoniae Isolates Uncovers Its Potential Association With Antibiotic Susceptibility. *Front Microbiol* 9**,** 1595. doi: https://doi.org/10.3389/fmicb.2018.01595.

Poirel, L., Dortet, L., Bernabeu, S., and Nordmann, P. (2011). Genetic features of blaNDM-1-positive Enterobacteriaceae. *Antimicrob Agents Chemother* 55**,** 5403-5407. doi: https://doi.org/10.1128/AAC.00585-11.

Poirel, L., Heritier, C., Tolun, V., and Nordmann, P. (2004). Emergence of oxacillinase-mediated resistance to imipenem in Klebsiella pneumoniae. *Antimicrob Agents Chemother* 48**,** 15-22. doi: https://doi.org/10.1128/AAC.48.1.15-22.2004.
